# Cordycepin suppresses growth and virulence of *Magnaporthe oryzae* via mitochondrial function and carbonic anhydrase-associated nitrogen metabolism

**DOI:** 10.1080/21505594.2026.2654300

**Published:** 2026-04-22

**Authors:** Yuejia Dang, Yujia Li, Guohui Xu, Tingzhen Wang, Qi An, Fei Gao, Xiaojing Liang, Xinyue Ji, Qian Li, Liang Wang

**Affiliations:** a School of Life and Health, Dalian University, Dalian, China; b Institute of Modern Agriculture Research, Dalian University, Dalian, China; c Dalian Oceanic Traditional Chinese Medicine Research Institute, Dalian University, Dalian, China; d Liaoning Provincial Key Laboratory of Lipid Metabolism, Dalian University, Dalian, China; e School of Biological Engineering, Dalian Polytechnic University, Dalian, China

**Keywords:** Cordycepin, *Magnaporthe oryzae*, antifungal, nitrogen metabolism, carbonic anhydrase

## Abstract

Cordycepin, a nucleoside analog naturally produced by *Cordyceps* fungi, exhibits broad-spectrum biological activity and has been reported to inhibit both prokaryotic and eukaryotic microorganisms, although its antifungal mechanisms remain poorly understood. In this study, we systematically evaluated the inhibitory effects of cordycepin on the growth, development, and pathogenicity of *Magnaporthe oryzae*, the causal agent of rice blast, and further elucidated its mode of action through transcriptomic analysis and gene function validation. Cordycepin significantly inhibited hyphal growth and reduced both conidial production and germination in a dose-dependent manner. Moreover, it severely impaired appressorium formation and increased the frequency of malformed structures, ultimately leading to attenuated virulence. Transcriptome profiling revealed that cordycepin reprogrammed multiple metabolic and signaling pathways, including nitrogen metabolism, fatty acid metabolism, melanin and chitin biosynthesis, and protein kinase networks. Notably, two carbonic anhydrase genes, *MoCA1* and *MoCA5*, were significantly downregulated and identified as candidate genes responsive to cordycepin. Functional analysis showed that MoCA5 localizes to mitochondria and interacts with MoCA1; deletion of either gene resulted in disrupted mitochondrial membrane potential, reduced ATP synthesis, and decreased pathogenicity. Collectively, our results indicate that cordycepin suppresses the growth and virulence of *M. oryzae* through coordinated modulation of mitochondrial function and nitrogen metabolism – associated pathways. These findings provide new insights into the antifungal action of cordycepin and identify potential molecular components involved in fungal metabolic adaptation to cordycepin stress.

## GRAPHICAL ABSTRACT

**Figure:**
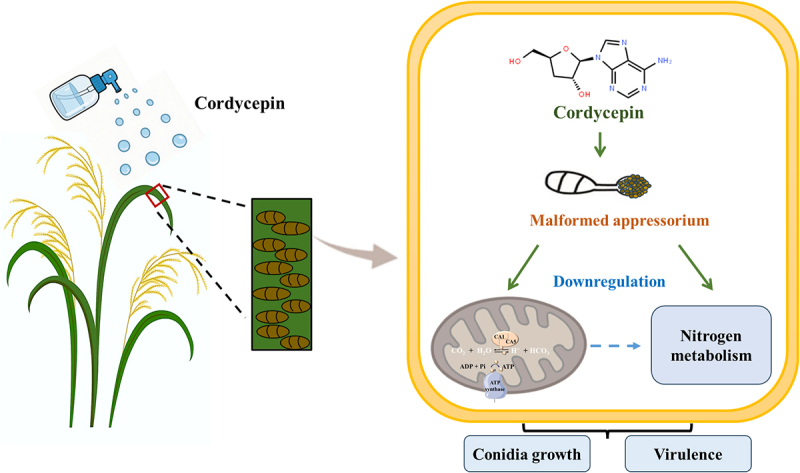

## Introduction

Rice blast, caused by the fungal pathogen *Magnaporthe oryzae*, is one of the most destructive diseases affecting rice production worldwide. It occurs in more than 85 rice-growing countries and poses a persistent and significant threat to rice cultivation, particularly in major rice-producing regions such as Asia and Africa. Global yield losses caused by rice blast are estimated to range from 10% to 30% annually, which is equivalent to the annual food supply for approximately 60 million people, thereby posing a serious challenge to global food security [1,2].

Infection by *M. oryzae* can result in a variety of symptoms, including leaf blast, panicle blast, node blast, and seedling blight. The infection process is highly complex and involves a sequential strategy comprising appressorium formation, penetration peg development, invasive hyphae growth, colonization, and secondary infection. During invasion, the pathogen secretes a wide array of effector proteins, such as AVR-Piz-t and MC69, which suppress host immune responses, disrupt reactive oxygen species (ROS) generation, interfere with signal transduction, and inhibit programmed cell death, thereby facilitating successful colonization. Moreover, *M. oryzae* can reprogram host metabolism by activating carbon and nitrogen metabolic pathways in rice cells, hijacking nutrients to support its own growth. The appressorium plays a crucial role in the infection process by accumulating high concentrations of glycerol and other polyols, generating an internal osmotic pressure of up to 8.0 MPa, which enables effective penetration through the plant epidermis [3,4].

The primary strategies for managing rice blast disease involve breeding rice varieties carrying resistance genes and applying of chemical fungicides [5]. Although chemical fungicides are widely used in plant disease control and crop protection, they face several significant challenges, including the rapid emergence of fungicide-resistant pathogen populations, a narrow spectrum of activity, and reliance on single-target modes of action. Furthermore, the long-term and large-scale application of these compounds has raised concerns about environmental risks and adverse impacts on non-target organisms, such as the substantial depletion of beneficial soil microbial and fungal communities [6,7].

Commonly used fungicides include tricyclazole, iprodione, pyraclostrobin, phenylmercuric acetate (PMA), and copper-based mixtures. For instance, tricyclazole inhibits the biosynthesis of dihydroxynaphthalene (DHN) melanin, thereby preventing appressorium maturation, and is primarily employed to control leaf and neck blast in rice. However, its potential toxicity, mutagenicity, and carcinogenicity pose risks to human health [8]. Iprodione disrupts fatty acid synthesis and fungal metabolism, but the emergence of resistant strains after prolonged use necessitates its rotation with other fungicides [9]. A mixture of PMA and copper-based fungicides has also shown efficacy in controlling rice blast, with relatively minor direct effects on human health and rice plants; nonetheless, pathogen resistance to this formulation has been increasingly reported [5]. Given these limitations, there is an urgent need to develop novel fungicides with unique mechanisms of action, multiple-target activity, and environmental compatibility to achieve sustainable and eco-friendly management of rice blast disease.

Cordycepin, also known as 3’-deoxyadenosine, is a natural nucleoside analog that was first isolated from the culture broth of *Cordyceps militaris* as early as the 1950 s. Although cordycepin is recognized as the first nucleoside antibiotic, it exhibits a wide range of physiological and pharmacological activities, including – but not limited to – antitumor activity [10], antioxidant effects [11], anti-inflammatory properties [12], and antiviral activity [13].

The mechanisms of cordycepin action in mammalian cells have been relatively well studied. It is generally accepted that cordycepin must first undergo phosphorylation to become active. Subsequently, it interferes with RNA metabolism by prematurely terminating RNA chain elongation, inhibiting polyadenylation at the 3’ end of mRNA, and disrupting key signaling pathways such as mTOR, PI3K/Akt, and AMPK. Cordycepin has also been shown to induce cell cycle arrest and apoptosis, and to suppress the expression of pro-inflammatory factors such as NF-κB, IL-6, and TNF-α.

In addition, cordycepin is considered a novel broad-spectrum antibiotic with demonstrated antibacterial properties. It has been shown to exert antibacterial and anti-inflammatory effects in *Helicobacter pylori*-infected mice [14]. It also causes substantial damage to the cytoplasmic membranes of *Escherichia coli* and *Bacillus subtilis*, and binds to bacterial genomic DNA, thereby disrupting cellular functions and ultimately leading to cell death [15]. Furthermore, 12 cordycepin derivatives have exhibited potent inhibitory activity against *E. coli* (strain 1924), *Staphylococcus aureus* (4220), and *Streptococcus mutans* (3289) [16]. Cordycepin also demonstrates antifungal activity, as evidenced by its efficacy in a mouse model of invasive candidiasis [17], suggesting its potential for development as a clinically relevant antifungal agent.

In the context of agricultural biological control, in silico analyses including molecular docking and protein modeling have revealed that cordycepin has a higher binding affinity for the β-chain 2 of tubulin in *Alternaria alternata* and *Alternaria mali* than that of the commercial fungicide tebuconazole, suggesting strong antifungal activity against these phytopathogens [18]. These findings highlight the potential of cordycepin as a promising biocontrol agent in agriculture.

To date, the reported antifungal mechanisms of cordycepin include disruption of cell membrane and cell wall integrity, inhibition of respiratory metabolism, and interference with DNA replication and transcription [19]. However, related studies remain limited in number and mechanistic depth, and there are currently no published reports on the use of cordycepin against *M. oryzae*, the causal agent of rice blast.

Therefore, this study systematically evaluates the inhibitory effects of cordycepin on *M. oryzae*, including its impact on hyphal growth, conidiation, and pathogenicity. Transcriptomic analyses revealed that cordycepin regulates multiple metabolic pathways and biological processes – including those related to energy metabolism – and identified key responsive genes along with their subcellular localization. These findings provide preliminary mechanistic insights and highlight potential molecular components involved in the cordycepin response, which may contribute to the development of novel and environmentally friendly strategies for rice blast control.

Furthermore, the elucidation of the cordycepin biosynthetic pathway [20] has enabled its efficient microbial production in engineered yeast strains. The establishment of microbial cell factories [21–23] for cordycepin synthesis paves the way for its practical and scalable application.

## Materials and methods

### Strains and plasmids

The *M. oryzae* wild-type strain JJ88 (provided by Professor Shi-Hong Zhang) was used in this study. It was originally isolated and purified from the *Oryza sativa* cultivar Jijing88 (Jilin Academy of Agricultural Sciences Rice Research Institute), a widely cultivated rice variety in Jilin Province, China. The ∆*MoCA5* strain, in which the *MoCA5* gene was deleted from JJ88, was constructed with the vectors pXEH 2.0 in our laboratory (Figure 4(a)), refering to previous research methods [24]. The ∆*MoCA5*/*CA5* strain represents the complementation of the *MoCA5* gene in the ∆*MoCA5* background.

*Escherichia coli* DH5α was used for all vector plasmid construction. The vectors pGADT7 and pGBKT7 were used in yeast two-hybrid assays, with *Saccharomyces cerevisiae* Y2H Gold (Δ*leu*, Δ*trp*, Δ*his*, Δ*ade*) as the host strain. The plasmid pKD7-Red, encoding a red fluorescent protein, was used for subcellular localization experiments. YN/YC vectors (pEarleyGate201/202) were employed for Gateway seamless cloning. AGL-1 strain was used for transformation of *M. oryzae*. *Agrobacterium tumefaciens* strain GV3101 (pSoup p19) was used for transformation of *Nicotiana benthamiana*. All plasmids were maintained in our laboratory or obtained as a kind gift from Professor Zhang’s research team.

### Media and culture conditions

*E. coli* was cultured in LB medium (10.0 g·L^−1^ tryptone, 5.0 g·L^−1^ yeast extract, 10.0 g·L^−1^ sodium chloride, pH 7.0 ± 0.1), supplemented with 50 μg·mL^−1^ kanamycin and 20 μg·mL^−1^ rifampicin when necessary.
*M. oryzae* was grown on Potato dextrose agar (PDA), complete medium (CM), or oatmeal tomato agar (OMA) plates. The compositions of these media were as follows:
PDA medium: 12.0 g·L^−1^ potato extract powder, 20.0 g·L^−1^ glucose, 14.0 g·L^−1^ agar, adjusted to pH 5.6 ± 0.2.
CM medium: 10 g·L^−1^ glucose, 1 g·L^−1^ Ca(NO_3_)_2_·4 H_2_O, 1 g·L^−1^ yeast extract, 0.5 g·L^−1^ enzymatic hydrolyzed casein, 0.5 g·L^−1^ acid hydrolyzed casein, 0.2 g·L^−1^ KH_2_PO_4_, 0.25 g·L^−1^ MgSO_4_·7 H_2_O, 0.15 g·L^−1^ NaCl, and 16–18 g·L^−1^ agar.
OMA medium: 30.0 g·L^−1^ oatmeal, 15.0–20.0 g·L^−1^ agar.
Cordycepin (Solarbio, China) was added to the media when required, at final concentrations ranging from 0.01 to 1.00 g·L^−1^.

All *M. oryzae* strains were routinely maintained on CM agar plates and preserved on filter paper at −20 °C. For conidiation assays, strains were inoculated onto OMA plates and incubated at 28 °C for 7 days in the dark, followed by 3 days of continuous illumination under fluorescent light [25]. Aerial hyphae were then removed by washing with sterile distilled water, followed by continued dark incubation for another 3 d.

### Assays for conidial production, growth, and development

The wild-type strain was grown on OMA medium supplemented with different concentrations of cordycepin. After 3 d of incubation at 28 °C, hyphae were gently washed off with sterile water. Small agar blocks were transferred to microscope slides and incubated in a moist chamber. Observations were made under a Nexcope NE620 microscope at 24, 48, and 72 h. At 72 h, fungal structures were stained with lactophenol cotton blue. Conidia were collected and counted using a hemocytometer. All assays were conducted in triplicate and repeated three times independently.

For assays of conidial germination and appressorium formation, conidial suspensions (1 × 10^5^ /mL) were applied to hydrophobic coverslips, and 100 μL of cordycepin solution at various concentrations was added around the drop. Observations were recorded at 1, 2, 3, 4, and 6 h. Each experiment included three replicates per strain and was repeated three times. The appressoria were also dehydrated using a graded ethanol series and then observed for morphological characteristics using a scanning electron microscope (Hitachi S-4800 SEM). For turgor pressure, 2–4 M glycerol was used to treat the appressoria for 3 min at 14 and 24 hpi, and at least 100 appressoria were counted to observe their collapse rate [26].

The ∆*MoCA5* and *∆MoCA5*/*CA5* strains were analyzed using the same procedures, except that no cordycepin was added; instead, 100 μL of sterile water was used as a control.

### Rice sheath penetration and plant infection assays

To assess the effect of cordycepin on the pathogenicity of *M. oryzae*, conidia from wild-type strains were harvested from OMA medium as described above. Three infection strategies were employed:

- Direct conidial spraying: Rice seedlings (*Oryza sativa* cv. Lijiangxintuanheigu) were sprayed with 2 mL of a conidial suspension (5 × 10^4^ conidia/mL in 0.2% gelatin) prepared from OMA medium supplemented with various concentrations of cordycepin.
- Post-infection spraying: Rice seedlings were first sprayed with a conidial suspension (same concentration as above) collected from OMA medium without cordycepin, followed by spraying with cordycepin solutions of varying concentrations after a short interval.
- Wound inoculation: Rice leaves were uniformly scratched, and mycelial blocks cultivated on OMA containing different concentrations of cordycepin were applied directly onto the wounds.

After inoculation, all plants were incubated in the dark at 28 °C for 48 h, and subsequently transferred to a growth chamber with a 16-hour light/8-hour dark photoperiod. Disease symptoms were assessed 7 d post-inoculation using the second method.

To observe early infection stages, inoculated leaves were sampled at 12 h post-inoculation (hpi). Conidia adhering to leaf surfaces were observed under a light microscope, and leaf segments containing spores were excised and fixed in 2.5% glutaraldehyde at 4 °C overnight. After washing and dehydration, conidial morphology was examined using a scanning electron microscope (S-4800, Hitachi High-Technologies Corporation, Japan).

For rice sheath inoculation, 100 μL of conidial suspension (5 × 10^4^ conidia/mL) collected from cordycepin-treated OMA medium was injected into detached rice leaf sheaths, which were then incubated in a humid chamber. Lesion development and necrosis were recorded upon leaf expansion. Invasive hyphal growth was quantified from 100 germinated conidia at 12, 24, and 48 hpi under a microscope (Nexcope NE620). Each treatment was performed in triplicate and repeated independently three times.

For the ∆*MoCA5* and ∆*MoCA5*/*CA5* strains, only the first infection method was used, and no cordycepin was added to the culture medium.

### Quantitative real-time PCR (qRT-PCR)

Total RNA was extracted from 7-day-old fungal mycelia cultured on PDA plates using the Spin Column Fungal Total RNA Purification Kit (Sangon Biotech). For cDNA synthesis, 2 μg of total RNA per sample was reverse transcribed using All-in-One 5× RT MasterMix. Quantitative real-time PCR was conducted using the CFX96 Optical Reaction Module (BIO-RAD, Singapore) with BlasTaq™ 2× qPCR MasterMix (Applied Biological Materials Inc.).

Relative gene expression levels were calculated using the 2^−ΔΔCq^, where Cq = Cq_gene_−Cq_actin_ [27]. The *M. oryzae* actin gene (*MGG_03982.6*) served as the internal reference for normalization. Each sample was analyzed in three technical replicates. Primer sequences used for qRT-PCR are listed in Table S1.

### Subcellular localization of MoCA5

To determine the subcellular localization of MoCA5, the gene was fused to a red fluorescent protein (RFP) tag via the *Sma* I site in vector pKD7-Red. A recombinant plasmid expressing MoCA5-RFP was introduced into the ∆*MoCA5* background strain to generate a complemented transformant (*pKD7-MoCA5::RFP*).

Fluorescent microscopy was used to observe the localization of MoCA5 in vegetative hyphae and conidia after 6 days of culture. To visualize mitochondria, hyphae and conidia were stained with 1 mM MitoTracker Green (Shanghai Yuanye Biotechnology Co., Ltd.) for 30–120 minutes at 37 °C. Images were captured using a laser scanning confocal microscope (FluoView FV3000, Olympus, Japan).

### ATP content assay

ATP levels in *M. oryzae* mycelia were measured using a commercial ATP Content Assay Kit (Nanjing Jiancheng Bioengineering Institute, China) following the manufacturer’s instructions, to assess the variations in ATP production among different strains.

### Yeast two-hybrid assay

The full-length cDNA of *MoCA5* was cloned into the pGADT7 vector as the prey construct, and the full-length cDNA of *MoCA1* was cloned into the pGBKT7 vector as the bait construct. Primer sequences are listed in Table S1. The two constructs were co-transformed into the yeast strain Y2H Gold. Transformants were initially selected on synthetic defined (SD) medium lacking leucine and tryptophan (SD−Leu−Trp), and then transferred to more stringent selective media: SD−Leu−Trp−His and SD−Leu−Trp−His−Ade. The positive interaction control was the co-expression of pGBKT7-53 and pGADT7-T, while the negative control was pGBKT7-Lam and pGADT7-T.

## Bimolecular fluorescence complementation (BiFC) assay

For BiFC analysis, the *MoCA1* gene fragment was cloned into the YN vector (pEarleyGate201) using Gateway seamless cloning to generate MoCA1-YN, while the *MoCA5* gene fragment was cloned into the YC vector (pEarleyGate202) to generate MoCA5-YC. These recombinant plasmids were transformed into *A. tumefaciens* strain GV3101 (pSoup p19). Positive transformants were screened on LB agar plates containing 50 μg·mL^−1^ kanamycin and 20 μg·mL^−1^ rifampicin. The selected strains were cultured and mixed for agroinfiltration into *N. benthamiana* leaves. After infiltration, YFP fluorescence signals were detected using a laser scanning confocal microscope (FV3000, Olympus, Japan). Prior to imaging, leaf samples were stained with JC-1 dye to visualize mitochondrial membrane structures.

### Mitochondrial membrane potential detection

To assess mitochondrial membrane potential in wild-type and mutant strains (∆*MoCA1* and ∆*MoCA5*), fungal mycelia were cultured on PDA plates containing sterile coverslips until they were fully overgrown. The coverslips were gently rinsed with JC-1 staining buffer to remove residual medium and unattached hyphae. Then, 50 μL of JC-1 working solution was applied to each coverslip to uniformly cover the mycelia. Samples were incubated at 37°C for 20 min.

As a positive control, CCCP (carbonyl cyanide 3-chlorophenylhydrazone) was added to disrupt the mitochondrial membrane potential, followed by JC-1 staining under the same conditions. After incubation, red and green fluorescence signals were observed and recorded using a laser scanning confocal microscope (FV3000, Olympus, Japan), allowing visualization of membrane potential differences between strains.

### Transcriptome analysis

The wild-type *M. oryzae* strain was cultured on PDA medium supplemented with 0.00 g·L^−1^, 0.01 g·L^−1^, 0.50 g·L^−1^, and 1.00 g·L^−1^ cordycepin for 7 days prior to transcriptome sequencing. Sequencing and preliminary data processing were performed by Beijing Novogene Bioinformatics Technology Co., Ltd. (Beijing, China).

RNA integrity was assessed using the Agilent 2100 Bioanalyzer. mRNA containing poly (A) tails was enriched using oligo (dT) magnetic beads, then fragmented and reverse-transcribed into cDNA to construct sequencing libraries. Libraries were sequenced using the Illumina platform (NovaSeq). A suitable reference genome (http://fungi.ensembl.org/Magnaporthe_oryzae/Info/Index) was selected for alignment. Gene expression levels were quantified based on FPKM (fragments per kilobase of transcript per million mapped reads). Differential expression analysis between sample groups was performed using the DESeq2 package (version 1.20.0), with genes defined as differentially expressed if they met the threshold of |log_2_FC| ≥1.0 and *p* < 0.05.

Functional enrichment analysis of differentially expressed genes (DEGs) was conducted using Gene Ontology (GO) and Kyoto Encyclopedia of Genes and Genomes (KEGG) annotations, and the results were visualized using appropriate bioinformatics tools.

### Statistical analysis

All experiments were conducted with a minimum of three biological replicates. Colony diameters were measured using ImageJ software. Statistical analyses of germination rates and relative gene expression levels were performed using SPSS Statistics 22 and Origin 2024. Data are presented as mean ± standard deviation (SD). Error bars in graphs represent SD. One-way ANOVA was used for statistical comparisons.

Statistical significance was determined as follows:

ns, not significant (*p* > 0.05); *, significant (*p* < 0.05); **, highly significant (*p* < 0.01); ***, extremely significant (*p* < 0.001).

## Results

### Cordycepin inhibits the growth and development of M. oryzae hyphae and conidia

**Figure 1.**
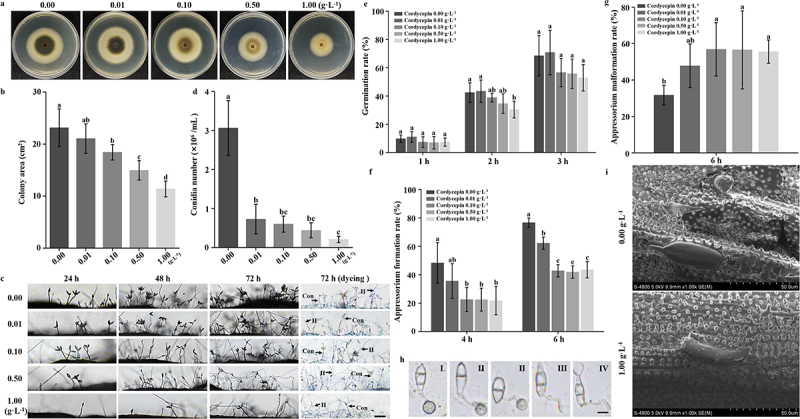
Cordycepin significantly impairs the growth and development of *M. oryzae* hyphae and conidia. (a) *M. oryzae* was cultured on PDA medium at 28 °C for 7 days under different concentrations of cordycepin to assess mycelial growth. (b) Colony areas were measured and analyzed statistically. (c) Conidiophore development was observed at 24, 48, and 72 h post-treatment with varying concentrations of cordycepin. At 72 h, conidiophores were stained with lactophenol cotton blue. Hyphae (H), appeared blue, and conidiophores (Con) appeared gray. Scale bar = 50 μm. (d) Quantification and statistical analysis of conidial production under different cordycepin concentrations. (e) Conidial germination rates were assessed microscopically at 1, 2, and 3 h. (f) Appressorium formation rates were evaluated microscopically at 4 and 6 h. (g) Appressorium malformation rates were recorded at 6 h post-inoculation. (h) Representative images of normal and malformed appressoria. I: normal appressorium; II – IV: malformed appressoria. Scale bar = 10 μm. (i) Scanning electron microscopy (SEM) was used to examine conidial morphology under treatments of 0 and 1.0 g·L^−1^ cordycepin. All data were analyzed using one-way ANOVA. Identical letters indicate no significant differences among groups, whereas different letters denote statistically significant differences. Each treatment was conducted in three independent biological replicates, each with three technical replicates. Error bars represent the mean ± standard deviation (SD).

To investigate the effects of cordycepin on *M. oryzae*, we examined hyphal growth, conidial germination, and appressorium formation under varying concentrations of cordycepin. As demonstrated in Figure 1(a,b), cordycepin suppressed hyphal growth in a concentration-dependent manner. The highest concentration (1.0 g·L^−1^) resulted in an approximately 50% reduction in colony area compared to the control.

As shown in Figure 1(c,d), the number of conidiophores and conidia formed at 24, 48, and 72 h post-inoculation was significantly lower in the treatment groups than in the control. Lactophenol cotton blue staining further confirmed that conidiophores were more abundant in the control group than in the cordycepin-treated groups. At 72 h, conidial yield in all cordycepin treatments was markedly reduced relative to the control (Figure 1(d)).

The appressorium is a specialized infection structure essential for host penetration and disease establishment by *M. oryzae* [28]. To evaluate the effect of cordycepin on conidial germination and appressorium formation, conidia were cultured in media with different concentrations of cordycepin and observed over time (Figure S1). Although conidial germination was largely unaffected except at the highest concentration at 2 h (Figure 1(e)), appressorium formation was significantly inhibited. After 6 h, the appressorium formation rate in all treatment groups decreased by approximately 40% compared to the control (Figure 1(f)). Notably, cordycepin induced appressorium malformation. Morphological abnormalities were frequently observed in treated groups (Figure 1(g,h)). This result was further validated by scanning electron microscopy (SEM), which revealed that conidia exposed to 1.0 g·L^−1^ cordycepin were deformed and failed to form appressoria (Figure 1(i)).

In conclusion, cordycepin significantly inhibits the hyphal growth of *M. oryzae* and disrupts conidium development by impairing both germination and appressorium formation. Moreover, it markedly increases the incidence of malformed appressoria, further impairing the pathogenic potential of the fungus.

### Cordycepin inhibits the pathogenicity of M. oryzae

**Figure 2.**
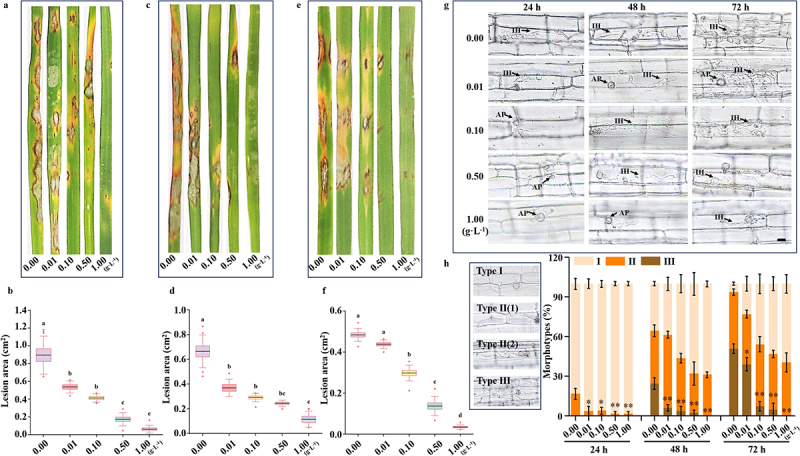
Cordycepin significantly reduces pathogenicity and inhibits invasive hyphal growth of *M. oryzae*. (a) Spray inoculation assay on rice leaves. A conidial suspension (5 × 10^4^ conidia/mL in 0.2% gelatin) was applied, followed by foliar treatment with various concentrations of cordycepin. (b) Lesion areas on rice leaves were quantified. (c) Spray inoculation using conidia collected from cultures treated with different cordycepin concentrations. (d) Statistical analysis of lesion areas from c. (e), hyphal inoculation assay on rice leaves using mycelia grown under various cordycepin concentrations. (f) Lesion area quantification corresponding to e. All experiments were independently repeated three times. One-way ANOVA was used for statistical comparisons; groups sharing the same letter are not significantly different, whereas different letters indicate significant differences. Error bars represent mean ± sd. (g) Rice sheath infection assay. Representative images of invasive hyphae at 12, 24, and 48 h post-inoculation (hpi). Scale bar = 10 μm. IH, invasive hyphae; AP, appressorium. (h) Infection types were classified into three categories. Type I, appressoria formed but no penetration occurred; type II, invasive hyphae exhibited one or two branches without spreading to adjacent cells; type III, invasive hyphae developed multiple branches and extended into neighboring cells. At each time point (12, 24, and 48 hpi), more than 100 germinated conidia per sheath were scored. Statistical analysis was performed using independent sample *t*-tests (*p < 0.05, **p < 0.01). Error bars represent mean ± sd from three independent experiments.

To further evaluate the effect of cordycepin on the pathogenicity of *M. oryzae*, we first inoculated rice leaves with untreated conidia and subsequently applied varying concentrations of cordycepin to the infected foliage. The results demonstrated a concentration-dependent reduction in lesion size on rice leaves. Notably, at a concentration of 1.0 g·L^−1^, the leaves exhibited almost no visible disease symptoms (Figure 2(a,b)).

Additionally, conidia and hyphae pre-cultured in cordycepin-supplemented media showed significantly reduced virulence, resulting in smaller lesions on rice leaves (Figure 2(c–f)). To investigate the development of invasive hyphae (IH) within host cells, conidia pre-treated with different concentrations of cordycepin were injected into rice leaf sheaths, and histological observations were conducted at 24, 48, and 72 h post-inoculation (hpi). Microscopic analysis revealed a marked suppression of IH development in response to higher cordycepin concentrations (Figure 2(g)). Infection severity was further quantified by categorizing infection types. Statistical analysis showed that the infection levels in the 0.1, 0.5, and 1.0 g·L^−1^ treatment groups were significantly lower than those in the control group across all time points. Remarkably, no Type III infections were detected in the 1.0 g·L^−1^ group (Figure 2(h)).

These findings demonstrate that cordycepin significantly suppresses the pathogenicity of *M. oryzae* by inhibiting both conidial and hyphal infection processes, supporting the potential application of cordycepin as a novel, biologically-derived fungicide for the effective management of rice blast disease.

### Effects of cordycepin on the metabolic pathways of M oryzae: transcriptomic analysis

To gain deeper insights into the molecular mechanisms by which cordycepin inhibits the growth and pathogenicity of *M. oryzae*, we performed RNA sequencing (RNA-seq) on fungal hyphae treated with different concentrations of cordycepin (0, 0.01, 0.5, and 1.0 g·L^−1^). Differentially expressed genes (DEGs) were identified using the criteria |log_2_FC| ≥1.0 and *p* < 0.05 based on FPKM values. Functional enrichment analysis was conducted through KEGG and GO databases to determine key genes and metabolic pathways responsive to cordycepin.

**Figure 3.**
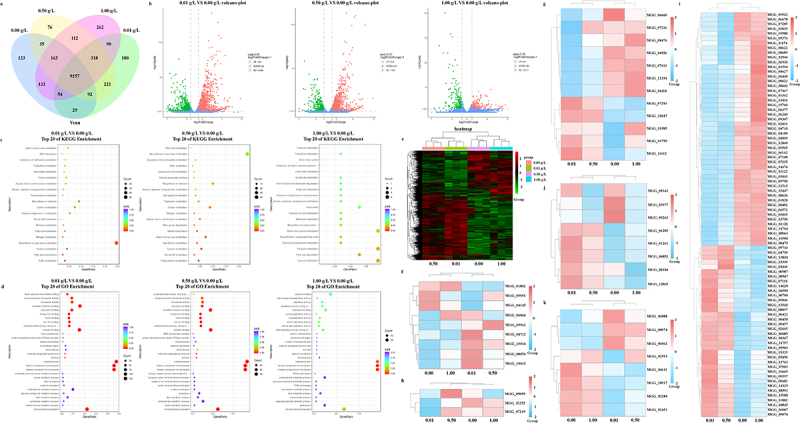
Transcriptomic profiling of *M. oryzae* in response to cordycepin treatment. (a) Venn diagram showing the overlap of expressed genes among treatment groups. (b) Volcano plots displaying the distribution of significantly upregulated and downregulated genes at different cordycepin concentrations. (c) KEGG pathway enrichment analysis of the top 20 significantly altered pathways. (d) Gene ontology (GO) enrichment analysis of the top 20 significantly altered biological processes. (e) Clustered heatmap illustrating the expression patterns of differentially expressed genes (DEGs) across various cordycepin concentrations. (f) Heatmap of genes associated with chitin-related enzyme biosynthesis. (g) Heatmap of DEGs involved in fatty acid metabolism. (h) Heatmap of genes related to melanin biosynthesis. (i) Heatmap of genes involved in protein phosphorylation. (j) Heatmap of genes involved in conidiation. (k) Heatmap of DEGs involved in nitrogen metabolism.

Venn diagram analysis showed that 9,157 genes were commonly expressed across all treatment groups (Figure 3(a)). Compared with the control group, the 0.01 g·L^−1^ group showed 2,623 DEGs, including 1,691 upregulated and 932 downregulated genes. The 0.5 g·L^−1^ group exhibited 1,701 DEGs (1,219 upregulated and 482 downregulated), whereas only 475 DEGs were detected in the 1.0 g·L^−1^ group (244 upregulated, 231 downregulated) (Figure 3(b)). In the KEGG enrichment analysis, the top 20 commonly enriched pathways in the cordycepin-treated group compared to the control group included “starch and sucrose metabolism,” “tryptophan metabolism,” “alanine, aspartate, and glutamate metabolism,” “vitamin B6 metabolism,” and “tyrosine metabolism.” Among these, the 0.01 g·L^−1^ treatment group showed significant enrichment in the “starch and sucrose metabolism” and “ABC transporter” pathways. The 0.5 g·L^−1^ treatment group exhibited notable changes in the “ether lipid metabolism” and “secondary metabolite biosynthesis” pathways, whereas the 1.0 g·L^−1^ treatment group displayed significant alterations in “thiamine metabolism” and “tryptophan metabolism” pathways (Figure 3(c)). GO enrichment analysis further revealed that the top 20 commonly enriched functional categories in the cordycepin-treated groups included “coenzyme binding,” “oxidoreductase activity,” “cofactor binding,” “plasma membrane,” “cell periphery,” “cell wall,” “external encapsulating structure,” “membrane part,” “intrinsic component of membrane,” “integral component of membrane,” “lipid metabolic process,” and “transmembrane transport.” Specifically, the 0.01 g·L^−1^ treatment predominantly affected the “lipid metabolic process,” whereas the 0.5 g·L^−1^ and 1.0 g·L^−1^ treatments were significantly enriched in “proteolysis” (Figure 3(d)).

Collectively, the KEGG and GO enrichment results indicate that cordycepin modulates a broad spectrum of biological processes and metabolic pathways, with notable involvement of nitrogen metabolism, sulfur metabolism, and fatty acid biosynthesis. Interestingly, cluster analysis revealed that the gene expression profiles of the 0.01 and 0.5 g·L^−1^ groups were similar, whereas the 1.0 g·L^−1^ group was more closely aligned with the control group (Figure 3(e)). Nevertheless, the 1.0 g·L^−1^ group still exhibited distinct gene expression differences compared with the control, indicating that high-dose cordycepin treatment induces a unique transcriptional response.

Chitin is a major structural component of fungal cell walls and is temporally and spatially regulated during fungal development and infection [29]. We investigated the expression profiles of genes involved in chitin biosynthesis (Figure 3(f)). Among these, the chitinase MoChia1 (*MGG_08054*) plays a critical role in the growth and development of *M. oryzae*, and its interaction with a rice tetratricopeptide repeat protein can trigger host immune responses [29]. In response to cordycepin treatment, the FPKM values of the *MoChia1* gene were significantly increased compared to the control group, with the most pronounced induction observed at 0.01 g·L^−1^, where the expression was approximately twofold higher than that of the control.

Chitin is synthesized by chitin synthases (CHS1–7), which contain conserved chitin synthase (CHS) and transmembrane domains. In *M. oryzae*, the chitin synthases genes *MoCHS1–7* (corresponding to *MGG_01802*, *MGG_04145*, *MGG_09551*, *MGG_09962*, *MGG_13014*, *MGG_13013*, and *MGG_06064*) are known to participate in chitin synthesis in conidia of *M. oryzae*, conidiation, cell wall repair, appressorium formation, appressorium-mediated penetration, invasive growth, and pathogenicity [30]. Transcriptomic analysis revealed that the FPKM values of *MoCHS1–3* were decreased under different concentrations of cordycepin treatment, whereas those of *MoCHS4–7* were significantly upregulated. Notably, *MoCHS5* and *MoCHS6* showed marked increases in expression, and both genes are closedly associated with conidiation, appressorium formation, and pathogenicity [30]. In addition, the chitinase *MGG_04732*, which has been reported as an important antifungal drug target [31], exhibited significantly elevated expression levels following treatment with 0.01 g·L^−1^ and 0.5 g·L^−1^ cordycepin. Collectively, these results indicate that cordycepin markedly influences the expression of chitin biosynthesis- and chitin remodeling-related genes in *M. oryzae*, thereby modulating its developmental processes and pathogenicity.

During *M. oryzae* infection, lipids serve as major energy reserves and are critical determinants of fungal pathogenicity [32]. Accordingly, we analyzed the differential expression of genes involved in fatty acid metabolism (Figure 3(g)). Notably, most of these genes exhibited transcriptional changes in response to cordycepin treatment at concentrations of 0.01 g·L^−1^ and 0.5 g·L^−1^ compared with the control group. The fatty acid desaturase *Fad2* (*MGG_01985*), which participates in the biosynthesis of polyunsaturated fatty acid-derived phospholipases and positively regulates iron-dependent apoptosis associated with conidial development [33], showed markedly increased FPKM values following cordycepin treatment. This induction was particularly pronounced at 1.0 g·L^−1^. Similarly, the β-fatty acid synthase *FAS1* (*MGG_04118*), a key regulator of cell wall architecture, lipid biosynthesis, and lipid transport during hyphal growth [34], exhibited significant differential expression at 0.01 g·L^−1^ and 0.5 g·L^−1^ cordycepin concentrations relative to the control.

Melanin is indispensable for the pathogenicity of *M. oryzae*. The melanin layer in appressoria maintains cell wall rigidity, a prerequisite for generating the high turgor pressure required for host penetration [35]. We assessed the expression of genes related to melanin biosynthesis (Figure 3(h)) and found that *ALB1* (*MGG_07219*), *RSY1* (*MGG_05059*), and *BUF1* (*MGG_02252*) participate in the melanin synthesis pathway [36]. Notably, significant differences in the FPKM values of these genes were observed compared with the control group under different concentrations of cordycepin treatment, with the strongest suppression detected at 0.01 g·L^−1^, where all genes showed markedly reduced expression.

Protein kinases are central components of signaling networks that regulate fungal growth, differentiation, and pathogenicity [28]. Given that kinase inhibition is one of the primary mechanisms through which cordycepin acts in mammalian systems, we specifically investigated the expression of genes associated with protein kinase activity (Figure 3(i)). These genes displayed distinct expression patterns, with similar profiles observed at the intermediate concentrations (0.01 and 0.5 g·L^−1^) and clear differences relative to the high-concentration and control groups. Notably, *MGG_09565* and *MGG_08097* are linked to the MAPK signaling pathway of the pathogenic mitogen-activated protein kinase 1 (Pmk1) [28]. In addition, the protein kinase C (PKC1) (*MGG_08689*), a serine/threonine kinase, is indispensable for the survival of *M. oryzae* [37]. Under different concentrations of cordycepin treatment, these genes showed significant changes in FPKM values compared with the control group, indicating that cordycepin modulates protein kinase – related signaling pathways in *M. oryzae* in a concentration-dependent and multifaceted manner.

The formation of conidia plays a critical role in the infection cycle of fungal pathogens, and inhibition of conidiation at an early stage is considered an effective strategy for disease control from an epidemiological perspective [38]. Accordingly, we performed differential expression analysis of genes associated with conidial development (Figure 3(j)). Among these, *COS1* (*MGG_03977*) a putative transcriptional regulatory factor, is required for conidiophore formation and is essential for early conidiophore development [38]. Two additional genes involved in the induction of conidiation, *MGG_05343* and *MGG_09263*, are also implicated in conidial formation [39]. As shown in the Figure 3(j), the expression levels of these three genes were significantly reduced following cordycepin treatment. The homeobox genes *Mohox1* (*MGG_04853*), *Mohox2* (*MGG_00184*), *Mohox4* (*MGG_06285*), and *Mohox7* (*MGG_12865*) are known to participate in hyphal growth, conidial formation, and appressorium development. Notably, *Mohox2* and *Mohox7* function as essential homeobox transcription factors during conidial and appressorial development in *M. oryzae* [40]. Transcriptomic data show that *Mohox1* and *Mohox4* were significantly upregulated in the cordycepin-treated groups, while *Mohox2* and *Mohox7* were significantly increased at 0.01 g·L^−1^ and 0.5 g·L^−1^, but significantly decreased at 1.0 g·L^−1^. These results indicate that cordycepin regulates conidiation-related gene expression in a concentration-dependent manner.

**Figure 4.**
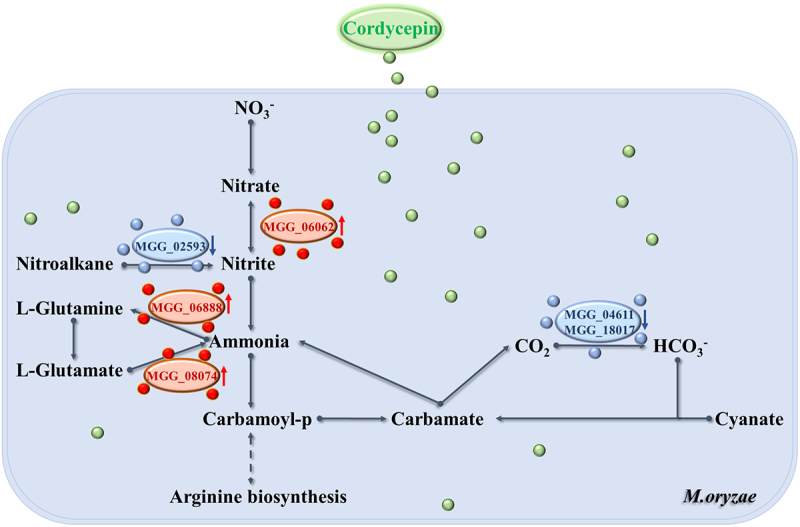
Nitrogen metabolism pathway in *M. oryzae* under cordycepin treatment. Expression changes of key genes involved in the nitrogen metabolism pathway following cordycepin treatment are illustrated. Red arrows indicate upregulated genes, while blue arrows represent downregulated genes.

In plant pathogenic fungi, nitrogen sources act as metabolic switches that regulate infection-related gene expression [41]. Consistent with this, several genes involved in nitrogen metabolism were differentially expressed among the cordycepin treatment groups (Figure 3(k)). Specifically, the FPKM values of *MGG_06888* (encoding glutamine synthetase), *MGG_08074* (encoding glutamate synthase), and *MGG_02593* (encoding nitroalkane synthase) were significantly upregulated following cordycepin treatment. In contrast, multiple other nitrogen metabolism-related genes exhibited marked downregulation. Comparative analysis revealed a consistent expression pattern of key nitrogen metabolism genes across all cordycepin-treated groups. Based on these findings, we propose a potential regulatory model of how cordycepin modulates the expression of nitrogen metabolism-related genes in *M. oryzae* (Figure 4). Cordycepin appears to enhance the conversion of nitrate to nitrite, followed by the reduction of nitrite to ammonia via nitrite reductase. The resulting ammonia is then utilized for synthesizing organic nitrogen compounds. During this process, the gene *MGG_06062* (encoding nitrate synthesis) is upregulated. Meanwhile, intracellular nitroalkanes may also contribute to nitrite production, during which *MGG_02593* (encoding nitroalkane synthesis) is downregulated. The nitrite is then converted into ammonia and used to synthesize L-glutamine, accompanied by the upregulation of *MGG_06888* (encoding glutamine synthetase). L-glutamine is converted into L-glutamate, which can release ammonia again, with *MGG_08074* (encoding glutamate synthesis) being upregulated in this cycle. Additionally, ammonia can participate in the synthesis of carbamoyl phosphate, which may enter the arginine biosynthesis pathway or be further transformed into carbamate. Carbamate is subsequently decomposed into ammonia and CO_2_. CO_2_ reacts with water to form HCO_3_^−^, during which *MGG_04611* (encoding carbonic anhydrase) and *MGG_18017* (encoding carbonic anhydrase) are downregulated. Finally, HCO_3_^−^ can react with cyanate esters in fungal cells to regenerate carbamate, completing the nitrogen cycle.

In summary, cordycepin reprograms nitrogen metabolism and regulates associated signaling pathways in *M. oryzae*, thereby significantly impairing fungal growth and pathogenicity. Notably, *MGG_18017* was downregulated to varying degrees (−1.7~-0.4-folds) under different concentration treatments, suggesting that it may serve as a candidate regulatory node through which cordycepin exerts its antifungal effects. Based on this observation, we subsequently conducted a comprehensive investigation of its subcellular localization and functional roles.

### MoCA5 is localized to the mitochondria and interacts with MoCA1

To elucidate the molecular mechanism underlying the inhibitory effect of cordycepin on *M. oryzae*, we selected *MGG_18017*, a gene that exhibited significant expression changes in the nitrogen metabolism pathway based on transcriptomic analysis, for functional and interaction studies. According to predictions from the UniProt database (https://www.uniprot.org/uniprotkb/G4NJP0/entry), this gene encodes a zinc-dependent β-carbonic anhydrase, which contains a carbonic anhydrase domain spanning amino acid residues 142–305 (Figure S3a). Through protein sequence comparison, it was found that the protein encoded by *MGG_18017* also has three highly conserved amino acid residues of β-carbonic anhydrase (two Cys and one His) involved in coordination, namely Cys26, His91, and Cys93 (Figure S3b). Therefore, we designated the gene product as *MoCA5*.

**Figure 5.**
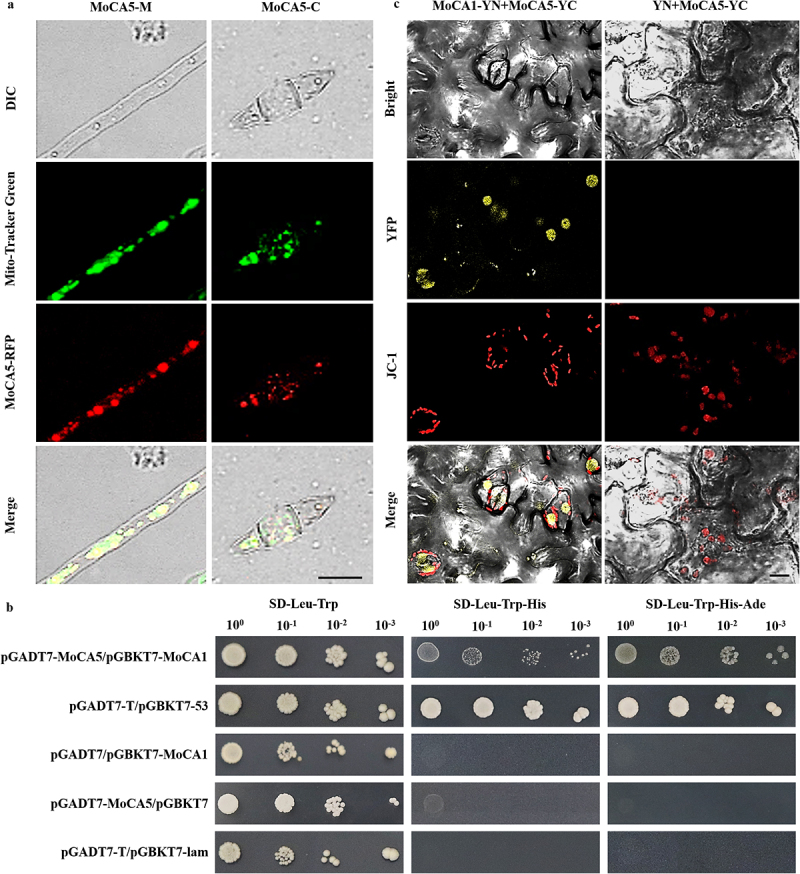
Subcellular localization and interaction of MoCA5 in *M. oryzae*. (a) Subcellular localization of MoCA5 in hyphae and conidia. Red fluorescence signals from MoCA5-RFP fusion protein in 6-day-old hyphae and conidia were detected via confocal microscopy and co-localized with mitochondria labeled by Mito-Tracker Green. Scale bar = 10 μm. (b) Yeast two-hybrid (Y2H) assay demonstrating the interaction between MoCA5 and MoCA1. The MoCA5-AD and MoCA1-BD constructs were co-transformed into yeast strain Y2H Gold and grown on SD−Leu−Trp (DDO), SD−Leu−Trp−His (TDO), and SD−Leu−Trp−His−Ade (QDO) selective media. Experimental group, pGADT7-MoCA5/pGBKT7-MoCA1; positive control, pGADT7-T/pGBKT7-53; negative control, pGadt7/pgbkt7-MoCA1, pGADT7-MoCA5/pGBKT7; blank control, pGADT7-T/pGBKT7-lam. (c) Bimolecular fluorescence complementation (BiFC) assay confirming the interaction between MoCA5 and MoCA1. Reconstituted YFP fluorescence signals were observed in *Nicotiana benthamiana* leaves co-expressing MoCA5-YC and MoCA1-YN, co-localizing with JC-1 staining of the mitochondrial membrane. MoCA5-YC and MoCA1-YN alone were used as negative controls. Scale bar = 5 μm.

To determine its subcellular localization, we constructed a transformant expressing *MoCA5* fused to red fluorescent protein (RFP) using molecular cloning and genetic transformation techniques (Figure S3c). Co-staining with Mito-Tracker Green revealed that *MoCA5* is specifically localized to the mitochondria in both hyphae and conidia, with higher expression observed in hyphal cells (Figure 5(a)).

In previous studies, another zinc-dependent β-carbonic anhydrase, *MoCA1*, was also reported to be localized to the mitochondria [25]. Given the similarity in subcellular localization and structural features between MoCA1 and MoCA5, we hypothesized that these two proteins might interact. To test this, we performed Y2H assays (Figure S3d) and BiFC assays (Figure S3e). The Y2H results confirmed the interaction. While both the experimental and negative control groups grew normally on double dropout (DDO) medium, only the experimental group and the positive control formed colonies on triple dropout (TDO) and quadruple dropout (QDO) media. Although the Y2H system may lack the sensitivity to fully detect the interaction, the growth pattern suggests that MoCA1 and MoCA5 interact under physiological conditions (Figure 5(b)). Furthermore, the BiFC assay provided additional evidence: when MoCA1-YN and MoCA5-YC were co-expressed in tobacco cells, fluorescence signals were observed in mitochondria, as indicated by JC-1 mitochondrial membrane staining. This indicates a direct interaction between MoCA1 and MoCA5 (Figure 5(c)).

Collectively, these findings offer new insights into the functional roles of β-carbonic anhydrases in *M. oryzae* and suggest that MoCA1 and MoCA5 May cooperatively function in mitochondria, potentially contributing to the antifungal activity of cordycepin.

### MoCA5 is required for the growth, development, and pathogenicity of M. oryzae

**Figure 6.**
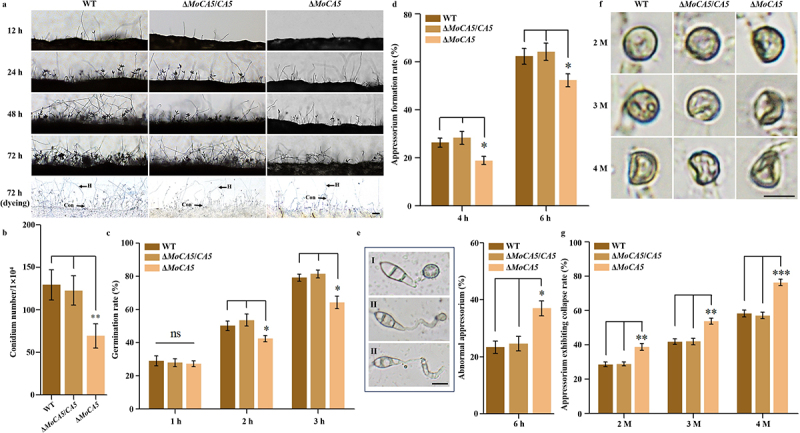
MoCA5 is essential for the growth and development of *M. oryzae*. (a) Conidiophore development was observed in wt, Δ*MoCA5*, and Δ*MoCA5*/*CA5* strains at 12, 24, 48, and 72 h. At 72 h, conidiophores were stained with lactophenol cotton blue. Hyphae (H) appear blue, and conidiophores (Con) appear gray. Scale bar = 50 μm. (b) Quantitative analysis of conidial production in wt, Δ*MoCA5*, and Δ*MoCA5*/*CA5* strains at 3 d post-inoculation. (c) Conidial germination rates were recorded at 1, 2, and 3 h using light microscopy. (d) Appressorium formation rates were assessed at 4 and 6 h. (e) Representative images of normal and abnormal appressoria at 6 h, and corresponding statistical analysis of the abnormal appressorium formation rate. Scale bar = 10 μm. (f) Observation for turgor pressure of the wt, Δ*MoCA5*/*CA5*, and Δ*MoCA5* strains was done in 2–4 M glycerol. Scale bar = 10 μm. (g) Appressorium collapse rate. Statistical significance was assessed using independent sample *t*-tests. ns, not significant (*p* > 0.05); *p < 0.05; **p < 0.01. Data represent means ± sd from three independent biological replicates.

To further elucidate the functional role of *MoCA5*, we constructed a gene knockout mutant (Δ*MoCA5*; Figure S4a) and a complemented strain (Δ*MoCA5*/*CA5*; Figure S3b) using molecular cloning and genetic transformation techniques. The Δ*MoCA5* mutant exhibited significantly reduced conidiophore and conidia production compared to the wild-type (WT) and complemented strains, with conidial yield decreased by approximately 50% at 3 d post-inoculation (Figure 6(a,b)).

Consistent results were observed in assays of conidial germination and appressorium formation (Figure S4b). The mutant also showed impaired conidial germination and appressorium formation (Figure 6(c,d)). Germination rates were significantly lower at 2 and 3 h, and appressorium formation was markedly reduced at 4 and 6 h. Furthermore, Δ*MoCA5* produced a significantly higher proportion of malformed appressoria (Figure 6(e)). Turgor pressure experiments revealed that the appressorium collapse rate of the knockout mutants was significantly higher by 15–25% in 2–4 M glycerol compared to the WT and the complemented strains (Figure 6(f,g)).

**Figure 7.**
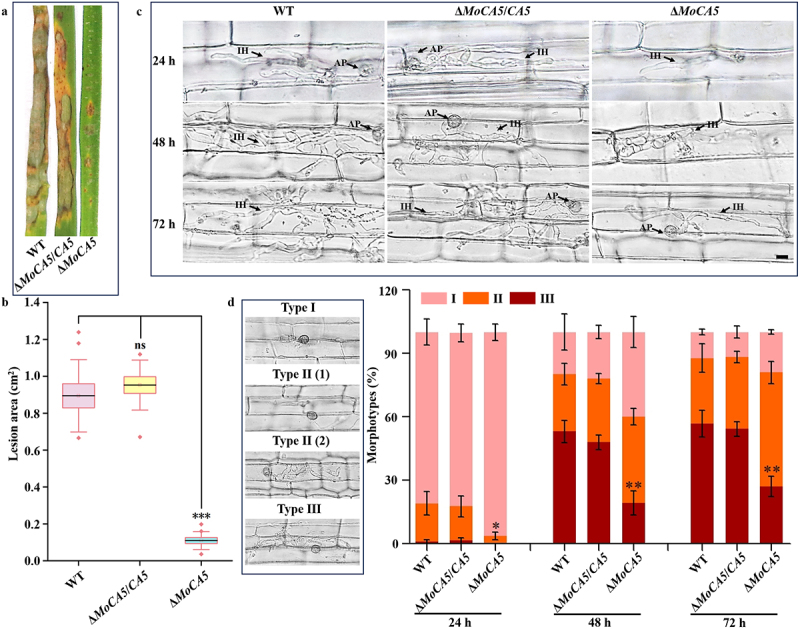
MoCA5 is required for full pathogenicity and invasive growth in *M. oryzae*. (a) Spray inoculation assay on rice leaves using conidial suspensions from wt, Δ*MoCA5*, and Δ*MoCA5*/*CA5* strains. (b) Quantitative analysis of lesion areas on infected rice leaves. (c) Microscopic observation of rice sheath infection at 24, 48, and 72 hpi. IH, invasive hyphae; AP, appressorium. Scale bar = 10 μm. (d) Classification of infection types, type I-appressorium formed without penetration; type II-limited IH development without cell-to-cell movement; type III-extensive IH branching with cell-to-cell invasion. All data are presented as means ± sd from three independent experiments, *p < 0.05; **p < 0.01.

To evaluate the contribution of *MoCA5* to pathogenicity, infection assays were conducted on rice leaves and leaf sheaths using conidial suspensions. The lesion areas caused by Δ*MoCA5* conidia were significantly smaller than those caused by the WT and complemented strains (Figure 7(a,b)). Microscopic examination of infected rice leaf sheaths revealed that invasive hyphal (IH) growth was markedly impaired in Δ*MoCA5* at 24, 48, and 72 hpi (Figure 7(c)). Additionally, the number of type III infection events in Δ*MoCA5* was less than half that observed in the control groups (Figure 7(d)).

Therefore, these findings demonstrate that MoCA5 plays an essential role in the conidiation, appressorium formation, and pathogenicity of *M. oryzae*.

### MoCA1 and MoCA5 are involved in the nitrogen metabolism of M. oryzae

Nitrogen metabolism plays a fundamental role in the growth, development, and environmental adaptation of both microorganisms and plants [42]. In our previous transcriptomic analysis, several genes potentially associated with nitrogen metabolism were identified, including *MGG_14061* (*MoCA1*), *MGG_18017* (*MoCA5*), *MGG_02593*, *MGG_06062*, *MGG_06888*, and *MGG_08074*. Among them, *MGG_06888* encodes a glutamine synthetase [43].

**Figure 8.**
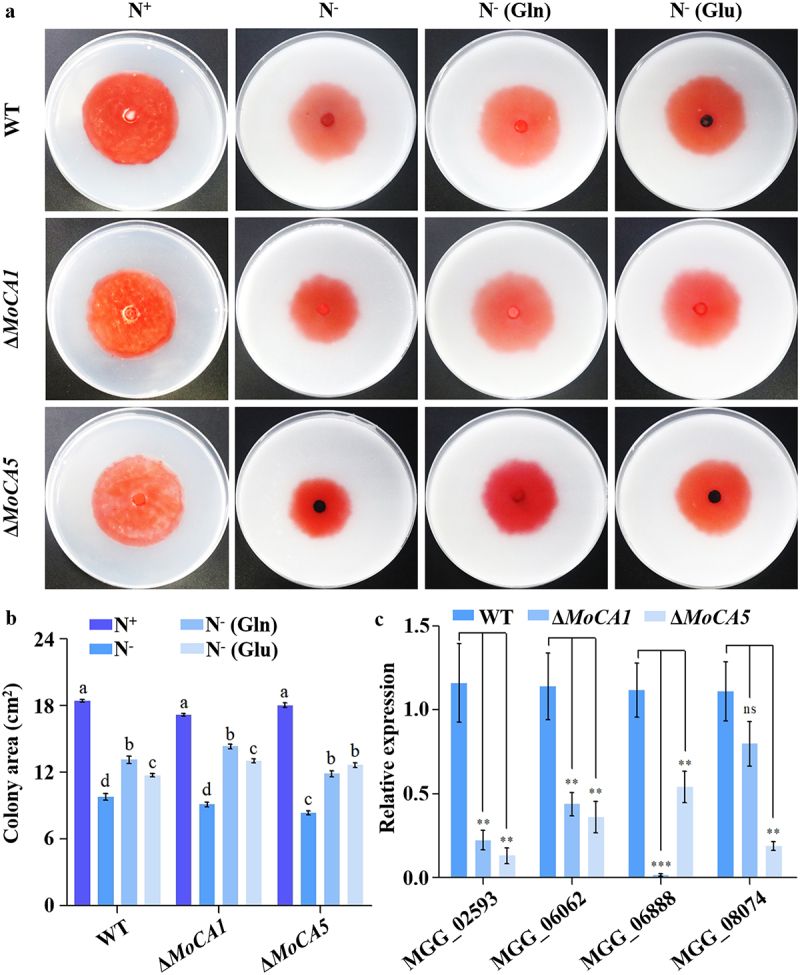
MoCA1 and MoCA5 are involved in the nitrogen metabolism of *M. oryzae*. (a) Colony morphology of wt, Δ*MoCA1*, and Δ*MoCA5* strains grown on complete medium (N^+^), nitrogen-deficient medium (N^−^), completely nitrogen-free with glutamine (N^−^(Gln)), completely nitrogen-free with glutamate (N^−^(Glu)). (b) Measurement and statistical analysis of colony areas under both nitrogen conditions. (c) Relative expression levels of nitrogen metabolism-related genes in wt, Δ*MoCA1*, and Δ*MoCA5* strains under standard culture conditions. Error bars indicate mean ± sd. Statistical analysis was performed using independent sample *t*-tests. ns, not significant (*p* > 0.05); *p < 0.05; **p < 0.01; ***p < 0.001.

To further investigate the roles of *MoCA1* and *MoCA5* in nitrogen metabolism, we cultured WT and the corresponding knockout mutants (Δ*MoCA1* and Δ*MoCA5*) on media supplemented with various nitrogen conditions: sufficient nitrogen (N^+^), completely nitrogen-free (N^−^), completely nitrogen-free with 0.2 mM glutamine (N^−^(Gln)), and completely nitrogen-free with 0.2 mM glutamate (N^−^(Glu)). Interestingly, under nitrogen-rich conditions, no significant differences in growth were observed between the strains. However, under nitrogen deprivation, both mutants exhibited markedly inhibited mycelial growth compared to the WT (Figure 8a). When glutamine (Gln) or glutamate (Glu) was added to the completely nitrogen-free medium, the growth capacity of the mutants was partially restored (Figure 8(a)). Quantitative analysis further confirmed a significant reduction in colony size in the mutant strains (Figure 8(b)).

In addition, we analyzed the expression of other nitrogen metabolism-related genes under normal growth conditions. The results revealed that the transcript levels of these genes were significantly downregulated in both Δ*MoCA1* and Δ*MoCA5* compared to the WT (Figure 8(c)).

Collectively, these results indicate that *MoCA1* and *MoCA5* contribute to glutamine-glutamate nitrogen metabolism not only through their individual activities but also by coordinating with other components of the nitrogen regulatory network. This coordination helps maintain metabolic balance and supports fungal growth and adaptation.

Compared with the wild-type (WT) strain, the Δ*MoCA1* and Δ*MoCA5* mutants exhibited an overall increased sensitivity to cordycepin stress (Figure S5), indicating that their functions of these genes or the metabolic pathways in which they are involved play a role in mitigating the toxic effects of cordycepin on *M. oryzae*. These results further imply that MoCA1 and MoCA5 are highly likely to act as downstream effector proteins in the cordycepin response.

### MoCA1 and MoCA5 participate in mitochondrial ATP synthesis

**Figure 9.**
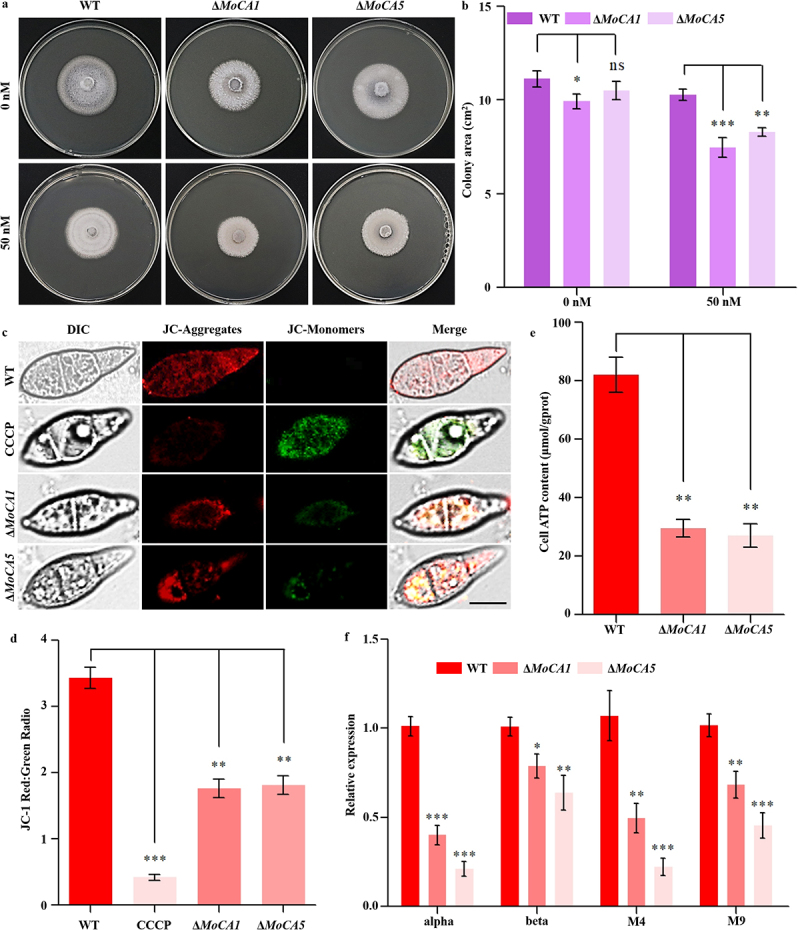
Roles of MoCA1 and MoCA5 in mitochondrial ATP synthesis in *M. oryzae*. (a) Colony morphology of wt, Δ*MoCA1*, and Δ*MoCA5* strains grown on PDA medium supplemented with 0 nM or 50 nM acetazolamide, a carbonic anhydrase inhibitor. (b) Quantification and statistical analysis of colony areas under different treatment conditions. (c) Assessment of mitochondrial membrane potential (MMP) using JC-1 staining. Confocal microscopy was used for imaging, with CCCP serving as a positive control. Scale bar = 10 μm. (d) Quantitative analysis of the red/green fluorescence intensity ratio reflecting MMP. (e) Intracellular ATP content measurement in wt, Δ*MoCA1*, and Δ*MoCA5* strains. (f) Relative expression levels of ATP synthase subunit genes (M4, M9, α, and β) determined by qRT-PCR. All experiments were independently repeated three times with three biological replicates per strain. Error bars indicate mean ± sd. Statistical significance was assessed using independent sample *t*-tests. ns, not significant (*p* > 0.05); *p < 0.05; **p < 0.01; ***p < 0.001.

Acetazolamide (Ace) is a small heterocyclic sulfonamide compound known to bind with high affinity to various carbonic anhydrases, acting as a potent carbonic anhydrase (CA) inhibitor [44]. In this study, WT, Δ*MoCA1*, and Δ*MoCA5* strains were cultured on PDA medium with or without 50 nM Ace. All three strains exhibited varying degrees of mycelial growth inhibition in the presence of Ace (Figure 9(a)), with the colony areas of Δ*MoCA1* and Δ*MoCA5* significantly smaller than that of the WT (Figure 9(b)).

Mitochondria are essential for cellular function, and the mitochondrial membrane potential (MMP) is a key indicator of mitochondrial activity [45]. To further explore the role of MoCA1 and MoCA5 in mitochondrial function, we used JC-1 staining to assess MMP. Confocal microscopy revealed that the red fluorescence of JC-aggregates in Δ*MoCA1* and Δ*MoCA5* was markedly reduced compared to WT, while green fluorescence from JC-monomers was almost undetectable (Figure 9(c)). Quantification of the red-to-green fluorescence ratio showed that WT maintained a significantly higher MMP than the mutants. CCCP, used as a positive control, drastically reduced this ratio, and both Δ*MoCA1* and Δ*MoCA5* strains exhibited ratios nearly half that of WT (Figure 9(d)), indicating impaired mitochondrial function in the mutants.

Given that ATP production is one of the primary functions of mitochondria, we next investigated whether the deletion of *MoCA1* or *MoCA5* affects intracellular ATP levels. Measurements revealed that ATP content in the WT strain was significantly higher than that in both Δ*MoCA1* and Δ*MoCA5* mutants (Figure 9(e)), indicating compromised ATP synthesis in the absence of either gene. To further explore the molecular basis of reduced ATP levels observed in the mutants, we examined the transcript levels of key subunits of the ATP synthase complex – subunit 4 (M4), subunit 9 (M9), subunit alpha (α), and subunit beta (β) [46]. Quantitative real-time PCR analysis revealed significant downregulation of all four genes in both Δ*MoCA1* and Δ*MoCA5* strains, with the most pronounced decrease observed in Δ*MoCA5* (Figure 9(f)).

These results indicate that *MoCA1* and *MoCA5* are indispensable for proper mitochondrial function in *M. oryzae*, playing a key role in sustaining ATP production likely by modulating the expression of ATP synthase subunits.

## Discussion

### Effects of cordycepin on the growth, development, pathogenicity, and potential mechanisms in M. oryzae

Cordycepin is a nucleoside analog structurally similar to adenosine, differing by the absence of a 3′-hydroxyl group on the ribose moiety [47]. It exhibits potent biological activity and is known to inhibit both bacterial and fungal growth. In this study, we demonstrated that cordycepin exhibits strong antifungal activity against *M. oryzae*, significantly suppressing hyphal growth and conidia development in a dose-dependent manner (Figure 1). The concentration range was selected to cover sublethal, intermediate, and inhibitory levels to capture dose-dependent transcriptional responses. Since appressorium formation from conidia is a critical step for *M. oryzae* to invade host rice cells, mainly through the generation of high turgor pressure within mature appressoria, we further assessed this structure. Cordycepin significantly suppressed appressorium formation and increased the proportion of morphologically aberrant appressoria, ultimately leading to a marked reduction in fungal pathogenicity (Figure 1 and 2). These findings suggest that cordycepin has considerable potential for controlling rice blast and may provide novel molecular targets for antifungal intervention.

To explore the molecular basis of the cordycepin response, we performed transcriptomic analyses combined with functional genetic experiments, including gene knockout and complementation. Among the differentially expressed genes, those related to chitinase drew our attention (Figure 3(f)). On one hand, chitin is a major structural component of fungal cell walls, and chitinases are involved in morphogenesis, cell division, autolysis, nutrient acquisition, and mycoparasitism. On the other hand, chitin also serves as a pathogen-associated molecular pattern (PAMP) that is recognized by plant immune receptors [29,48]. This suggests that cordycepin may interfere with host recognition of PAMPs, thereby modulating the plant’s defense response.

Previous studies in mammalian cells have shown that cordycepin disrupts intracellular kinase signaling pathways, including MAPK, AMPK, and mTOR, and inhibits DNA and RNA synthesis [14,49,50]. In *M. oryzae*, MAPK pathways regulate appressorium formation, penetration, cell wall integrity (CWI), and stress responses [51,52]. Phosphorylation signal reprogramming is crucial for proper appressorium development [28]. Here, transcriptomic results revealed altered expression of 85 protein kinases in response to different cordycepin concentrations (Figure 3(i)). To verify the transcriptomic reliability, six genes were selected for qPCR, and their expression patterns were consistent with RNA-seq data (Figure S2). Interestingly, hierarchical clustering showed that the 0.01 and 0.5 g·L^−1^ treatment groups shared similar expression profiles, whereas the 0 g·L^−1^ and 1.0 g·L^−1^ groups clustered together. This suggests that *M. oryzae* probably adopts distinct physiological strategies to cope with different levels of cordycepin stress. Additionally, melanin biosynthesis and fatty acid metabolism, two processes closely related to fungal virulence, were also disrupted by cordycepin (Figure 3(g,h)), further supporting its multifaceted antifungal activity [32,53].

Nitrogen is an essential element for all living organisms and forms the basis of growth and energy metabolism. The ability of fungi to utilize diverse nitrogen sources enables them to survive in various ecological niches and under nutrient-limited conditions. Fungi preferentially utilize high-quality nitrogen sources such as ammonium and glutamine, while genes involved in the utilization of secondary nitrogen sources are suppressed by nitrogen catabolite repression (NCR) [54]. In yeast, growth on poor nitrogen sources can be enhanced by upregulating enzymes for glutamate/glutamine biosynthesis and by increasing amino acid transporter activity. Nitrogen starvation or treatment with rapamycin, an mTOR inhibitor, leads to cell cycle arrest and stress responses [55]. In our study, transcriptomic data revealed significant changes in the expression of multiple nitrogen metabolism-related genes following cordycepin treatment (Figure 3(k)), suggesting that cordycepin may directly or indirectly perturb nitrogen metabolism pathways. The *MoCA1* and *MoCA5* knockout mutant strains exhibited a significantly enhanced response to cordycepin at concentrations of 0.01, 0.1, and 0.5 g·L^−1^ compared to the WT strain (Figure S5), further indicating that *MoCA1* and *MoCA5* are potential candidate genes for studying the cordycepin response. Interestingly, at a concentration of 1.0 g·L^−1^, all strains showed similar and significant suppression, suggesting that high concentrations of cordycepin exert a broad inhibitory effect on *M. oryzae* strains.

In this study, we recognize that the use of 1 g·L^−1^ cordycepin may not be feasible for large-scale agricultural applications. A major challenge is that cordycepin can be rapidly deaminated at the N6 position by adenosine deaminase in vivo, leading to its inactivation and reduced antifungal efficacy [56]. Consequently, higher concentrations are often needed to sustain antifungal activity, which increases production costs.

However, it is important to note that cordycepin is currently being explored as a lead compound for further structural modification and optimization to alleviate deamination and enhance stability [57,58]. Our research aims to investigate its pathways and cellular processes involved in the cordycepin response, providing a foundation for the development of more potent and economically viable derivatives. Future studies will focus on modifying the chemical structure of cordycepin to improve its stability, bioavailability, and antifungal potency, making it a more suitable candidate for agricultural applications.

### Functional characterization of the key gene MoCA5

In this study, we confirmed the mitochondrial localization of MoCA5 (Figure 5(a)) and its interaction with MoCA1 (Figure 5(b,c)). Although BiFC assays supported the interaction between MoCA1 and MoCA5, the yeast two-hybrid (Y2H) assay may have failed to detect the interaction due to insufficient sensitivity. Co-immunoprecipitation (Co-IP) is recommended for further validation. Previous research has demonstrated that MoCA1 localizes to mitochondria and plays a role in conidial development and pathogenicity in *M. oryzae* [25]. The expression pattern of *MoCA5* at various developmental stages suggests its involvement in fungal growth and development (Figure S4 (c)). Additionally, studies have shown that MoCA5 localizes to the mitochondria in both hyphae and conidia (Figure 5a). Nevertheless, further investigation is required to confirm the role of this protein during the infection process. Deletion of *MoCA5* resulted in reduced sporulation, lower germination rates, impaired appressorium formation, and attenuated virulence (Figure 6 and 7), indicating that *MoCA1* and *MoCA5* may function synergistically to support fungal growth and pathogenicity.

Mitochondria are central to cellular energy production, and their functional status directly affects ATP availability [59]. Cordycepin-induced cancer cell death has been associated with mitochondrial-mediated apoptosis, characterized by DNA fragmentation, TUNEL staining, cytochrome c release, and caspase activation [60]. In our study, we demonstrated that *MoCA1* and *MoCA5* are involved in maintaining mitochondrial function and ATP synthesis (Figure 9(c,d)). Interestingly, ATP levels were also significantly reduced in wild-type *M. oryzae* treated with cordycepin (Figure S4d), supporting the hypothesis that *MoCA1* and *MoCA5* may be involved in the cordycepin response, potentially through signaling pathways, acting as downstream effector proteins. Through mitochondrial dysfunction, induced apoptosis, and disruption of nitrogen metabolism, cordycepin ultimately impairs fungal growth and virulence. This study provides new insights into fungal stress responses to nutrient limitation and offers a theoretical basis for the development of novel antifungal agents and fungicide mechanisms.

Future work should include metabolomic profiling and functional studies of other upregulated or downregulated genes identified in the cordycepin-treated groups through gene knockout or overexpression. Fatty acid metabolism and de novo lipid synthesis involve a series of complex biochemical reactions that occur in both the cytoplasm and mitochondria. Mitochondrial carbonic anhydrases, which exhibit high catalytic activity, play a critical role in regulating intracellular bicarbonate ion homeostasis and catalyzing the rapid carboxylation of pyruvate and acetyl-CoA, leading to the formation of acetyl-CoA and malonyl-CoA, respectively. Accumulating evidence indicates that the inhibition of mitochondrial carbonic anhydrases disrupts the functional integrity of the pyruvate, fatty acid, and succinate metabolic pathways. Targeting the mitochondrial carbonic anhydrase isoforms has been shown to modulate gluconeogenesis and lipogenesis [61]. This represents the central focus of our ongoing research work. Additionally, exploring the *O. sativa*–*M. oryzae* interaction systemically could provide a more comprehensive understanding of the infection process and host response.

## Conclusion

Overall, this study demonstrates that cordycepin significantly inhibits the growth and pathogenicity of *M. oryzae* by modulating multiple metabolic and signaling pathways, including nitrogen metabolism, chitinase expression, melanin synthesis, kinase regulation, and conidiation. *MoCA1* and *MoCA5* were identified as candidate components in glutamine-glutamate nitrogen metabolism and mitochondrial function, where they may act synergistically to maintain nitrogen homeostasis and ATP production. These findings provide new insights into the antifungal mechanisms of cordycepin and contribute to a better understanding of fungal responses to metabolic stress. Moreover, *MoCA1* and *MoCA5* represent promising molecular components of the cordycepin response network and may serve as valuable entry points for future mechanistic studies aimed at developing novel strategies to manage rice blast disease These findings provide new insights into the antifungal mechanisms of cordycepin and contribute to a better understanding of fungal responses to metabolic stress.

## Supplementary Material

1_v_Supplementary Information.docx

## Data Availability

The data that support the findings of this study are openly available in Figshare at https://doi.org/10.6084/m9.figshare.30095521.v1, reference number [62]. The transcriptome dataset is available in the NCBI Sequence Read Archive under accession number PRJNA1322113 at https://www.ncbi.nlm.nih.gov/bioproject/1322113, reference number [63].
