# Supplementary material for: Cordycepin suppresses growth and virulence of Magnaporthe oryzae via mitochondrial function and carbonic anhydrase-associated nitrogen metabolism
Source: Virulence. 2026 Apr 22;17(1):2654300. doi: 10.1080/21505594.2026.2654300 (PMC13108354; doi:10.1080/21505594.2026.2654300)
Supplement: 1_v_Supplementary Information.docx [file KVIR_A_2654300_SM3878.docx]

**Supplementary Information**

**Cordycepin Suppresses Growth and Virulence of *Magnaporthe oryzae* via Mitochondrial Function and Carbonic Anhydrase-Associated Nitrogen Metabolism**

Yuejia Dang^a,b*^, Yujia Li^a*^, Guohui Xu^a,b^, Tingzhen Wang^a,b^, Qi An^a,b^, Fei Gao^a,b^, Xiaojing Liang^a,b^, Xinyue Ji^a,b^, Qian Li^a,c,d*^, Liang Wang^e*^

^a^School of Life and Health, Dalian University, Dalian 116622, China.

^b^Institute of Modern Agriculture Research in Dalian University, Dalian 116622, China.

^c^Dalian Oceanic Traditional Chinese Medicine Research Institute, Dalian 116622, China.

^d^Liaoning Provincial Key Laboratory of Lipid Metabolism, Dalian 116622, China.

^e^School of Biological Engineering, Dalian Polytechnic University, Dalian 116034, China.

Yuejia Dang, ORCID: <https://orcid.org/0000-0002-6398-8381>; Email: dangyuejia@dlu.edu.cn; Yujia Li, Email: liyujia@s.dlu.edu.cn; Correspondence Qian Li, ORCID: 0000-0001-8340-5035; E-mail: liqian@dlu.edu.cn; Liang Wang, ORCID: 0000-0002-3740-6421; E-mail: liangwang@dlpu.edu.cn; ^*^ These authors contributed equally to this work.

**Supplementary data**

**Table S1 Primers used in this study**

| **Primer name** | **Sequences (5’-3’)** | **Description** |
| --- | --- | --- |
| MoCA5_upF | CCGCTCGAGGTGCTTGCGTCTAATG | Upstream fragment of *MoCA5* for gene deletion |
| MoCA5_upR | GAAGATCTACTGTCCTGAAGGGTTA |  |
| MoCA5_downF | CGGGATCCATAGGTCACTGCGGATAA | Downstream fragment of *MoCA5* for gene deletion |
| MoCA5_downR | ACGCGTCGACTTCGGTCTTGCCTGAT |  |
| MoCA5_L-HYG-F | ACAGACTAACCTGCTCG | Confirming the deletion of *MoCA5* |
| MoCA5_L-HYG-R | AGTTTGCCAGTGATACA |  |
| MoCA5_gene-F | TCCCCCGGGATGTTTAAAATCCGCCTGTC |  |
| MoCA5_gene-R | TCCCCCGGGGGGGCCGCCCATCCTCGGTG |  |
| MoCA5_HYG-R-F | GCCCTTCCTCCCTT |  |
| MoCA5_HYG-R-R | GAGCGGCTTCAACG |  |
| MoCA5_AD_F | GGAGGCCAGTGAATTCATGTTTAAAATCCGCCTGTCCGC | *MoCA5* cDNA fragment for Y2H assay |
| MoCA5_AD_R | CGAGCTCGATGGATCCGGGGCCGCCCATCCTCGG |  |
| MoCA1_BD_F | CCGGAATTCATGGCTCAAAATCAGGATG | *MoCA1* cDNA fragment for Y2H assay |
| MoCA1_BD_R | CGCGGATCCGCGAGCCGTCATAGAGGCG |  |
| MoCA5_YC_F | TCCCCCGGGATGTTTAAAATCCGCCTGTC | Full-length ORF of *MoCA5* for fusion with C-terminal YFP tag |
| MoCA5_YC_R | TCCCCCGGGGGGGCCGCCCATCCTCGGTG |  |
| MoCA1_YN_F | CTAGTCTAGAATGGCTCAAAATCAGGATG | Full-length ORF of *Moca1* for fusion with N-terminal YFP tag |
| MoCA1_YN_R | ACGCGTCGACGCGAGCCGTCATAGAG |  |
| MoCA5_qF | CCGTCGGGATCTGGTTCTG | qRT-PCR for *MoCA5* |
| MoCA5_qR | GTTGATGCGTTGGCACTCG |  |
| MGG_02593_qF | GGGTCATCAGACACGC | qRT-PCR for MGG_02593 |
| MGG_02593_qR | TCCGGTACCAGCAAAT |  |
| MGG_06062_qF | GATGGGCAAGTCTACG | qRT-PCR for MGG_06062 |
| MGG_06062_qR | TTTCCGAGTGAATTGTC |  |
| MGG_06888_qF | GGCAGCATCGAGGAGTT | qRT-PCR for MGG_06888 |
| MGG_06888_qR | GCCGGTAATCTGGTAAGG |  |
| MGG_08074_qF | GGCAGAACAGCTCGCG | qRT-PCR for MGG_08074*5* |
| MGG_08074_qR | TGCTTCCGGCAACCAG |  |
| Alpha_qF | CGTGGTGAACGCTTGA | qRT-PCR for ATP synthase subunit alpha |
| Alpha_qR | TGAGGTGGGACAGGAA |  |
| beta_qF | TTTCCGATTTACTCAGGC | qRT-PCR for ATP synthase subunit beta |
| beta_qR | GGTGGTAATACGCTCTTG |  |
| M4_qF | TCATCAAGTTCGGTGGC | qRT-PCR for ATP synthase subunit 4 |
| M4_qR | TTGGAGACGGCAAAGAG |  |
| M9_qF | CGTGGTCAGCTTTTCA | qRT-PCR for ATP synthase subunit 9 |
| M9_qR | TGAGCAAGAAGGCAAC |  |

**Supplementary Figures**


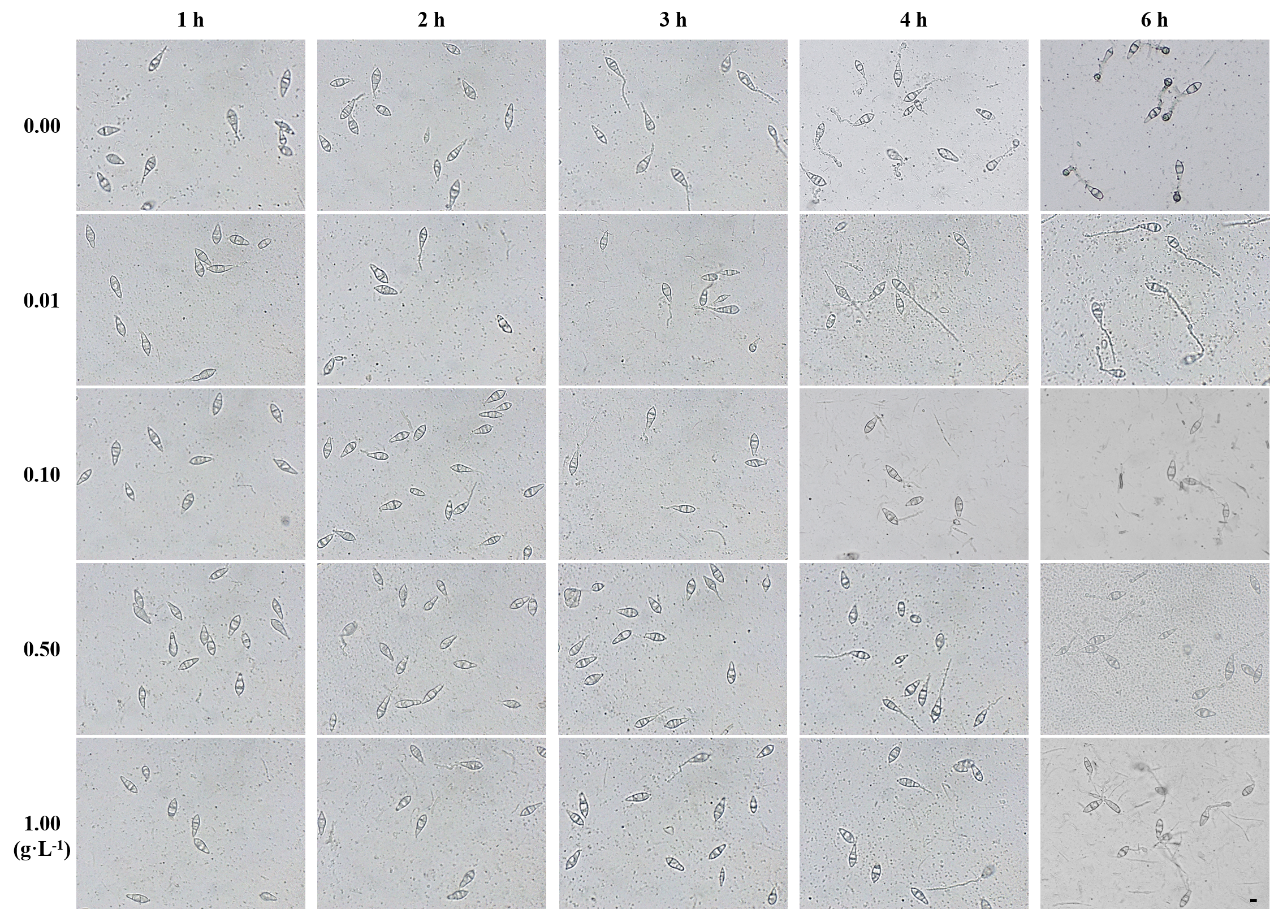


**Fig. S1.** Time-dependent effects of varying cordycepin concentrations on conidial germination in *M. oryzae*. Conidial suspensions of all strains were inoculated with different concentrations of cordycepin from 0-1.0 g·L⁻¹, observed at 1, 2, 3, 4, and 6 h. Bar = 10 μm.


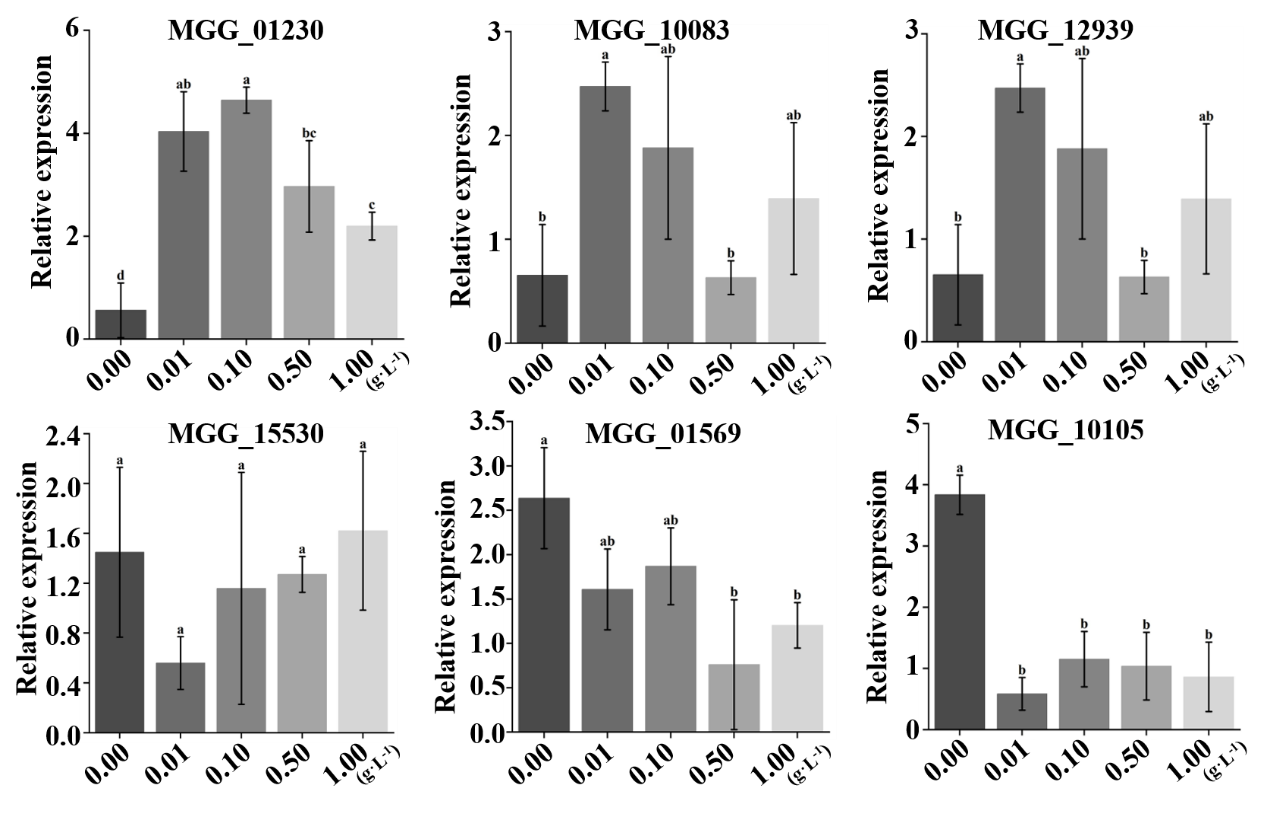


**Fig. S2.** Validation of reliability of the transcriptomic data by quantitative real-time PCR (qRT-PCR). *MGG_01230*, *MGG_10083*, *MGG_12939*, *MGG_15530*, *MGG_01569* and *MGG_10105* were used as the internal reference for normalization.


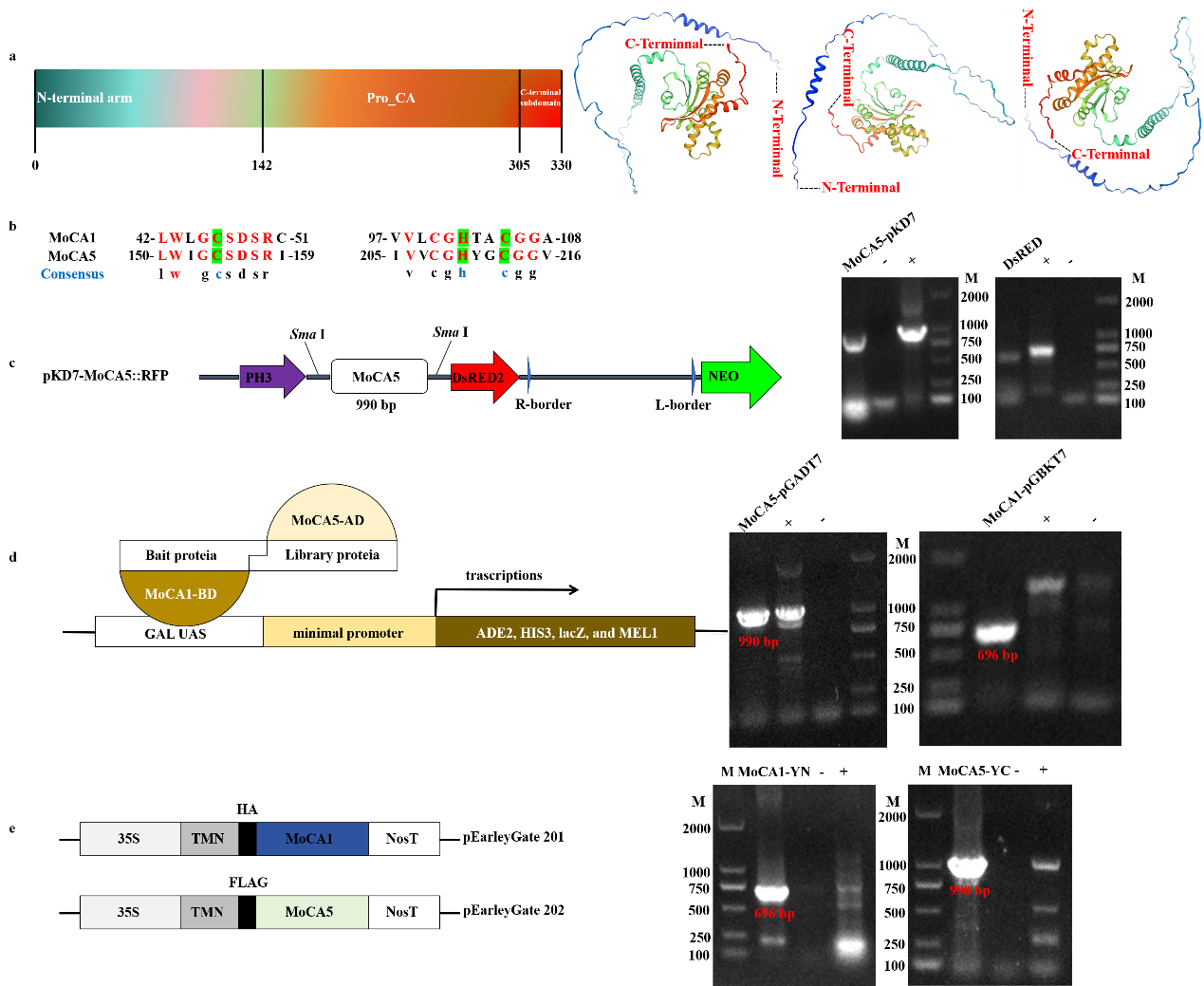


**Fig. S3.** Structural characterization, subcellular localization, and protein interaction analysis of *MoCA5*. (a) Predicted domain architecture and tertiary structure of MoCA5. (b) Sequence alignment. The zinc ion in MoCA1 and MoCA5 is coordinated by three highly conserved residues: two cysteines (Cys) and one histidine (His). (c) Construction of the subcellular localization vector and verification of gene integration. (d) Construction of yeast two-hybrid (Y2H) vectors and validation of gene insertion. (e) Diagram of vector construction for bimolecular fluorescence complementation (BiFC) assays and corresponding validation by gel electrophoresis.


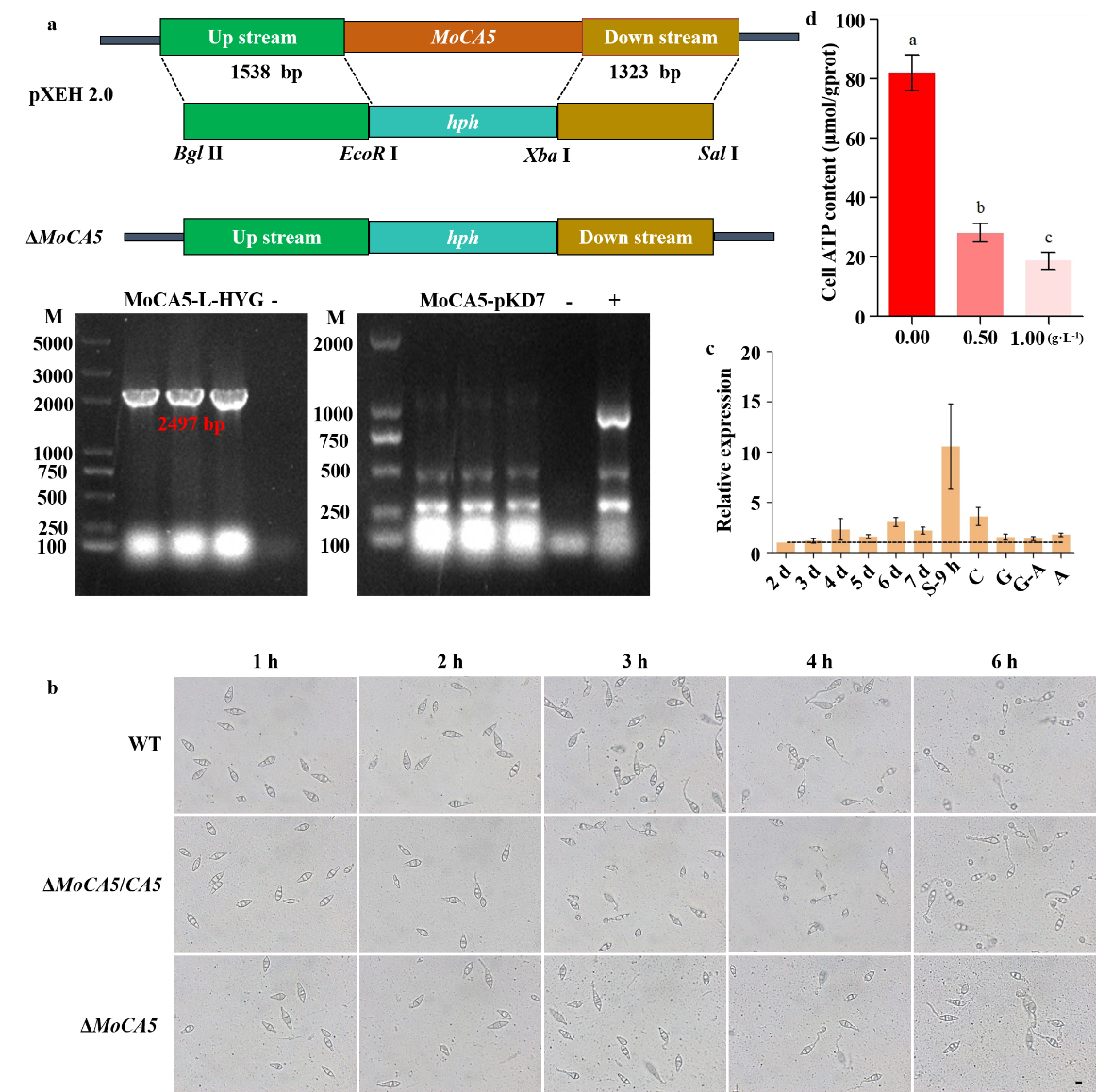


**Fig. S4.** Expression profile and functional validation of *MoCA5* and its role in energy metabolism under cordycepin treatment. (a) Schematic representation of the *MoCA5* gene knockout strategy and validation by gel electrophoresis. (b) Observation of conidial germination and appressorium formation on artificial hydrophobic surfaces at 1, 2, 3, 4, and 6 h post-inoculation. Scale bar = 10 μm. (c) Expression levels of *MoCA5* at different developmental stages: hyphae (2-7 d), conidiophores (S-9 h), conidia (C), germination (G), germ tube and appressorium formation (G–A), and mature appressorium (A). (d) Intracellular ATP content in *M. oryzae* hyphae treated with 0, 0.5, and 1.0 g·L⁻¹ cordycepin. Data were analyzed using one-way ANOVA. Bars with the same letters are not significantly different, while bars with different letters indicate significant differences between groups (*p* < 0.05). Each treatment was conducted with three independent biological replicates. Error bars represent mean ± standard deviation (SD).


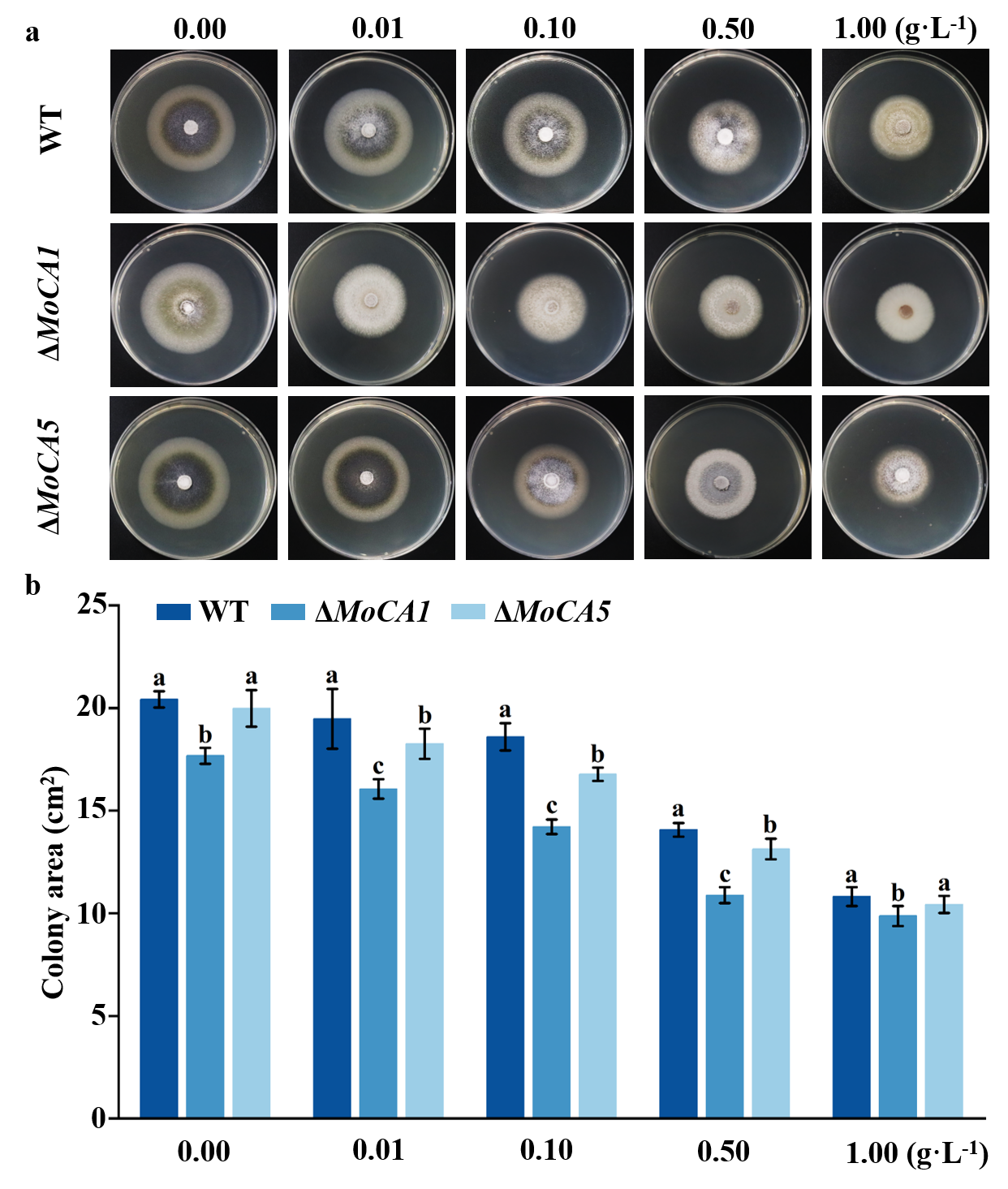
**Fig. S5.** Effects of different concentrations of cordycepin on mycelial growth of WT, Δ*MoCA1,* Δ*MoCA5*. (a) Colony morphology of WT, Δ*MoCA1,* Δ*MoCA5* on media containing different concentrations of cordycepin. (b) Colony area of WT, Δ*MoCA1,* Δ*MoCA5* on media containing different concentrations of cordycepin. Data were analyzed using one-way ANOVA. Bars with the same letters are not significantly different, while bars with different letters indicate significant differences between groups (*p* < 0.05). Each treatment was conducted with three independent biological replicates. Error bars represent mean ± standard deviation (SD).
